# Systematic review of basket trials, umbrella trials, and platform trials: a landscape analysis of master protocols

**DOI:** 10.1186/s13063-019-3664-1

**Published:** 2019-09-18

**Authors:** Jay J. H Park, Ellie Siden, Michael J. Zoratti, Louis Dron, Ofir Harari, Joel Singer, Richard T. Lester, Kristian Thorlund, Edward J. Mills

**Affiliations:** 1 Experimental Medicine, Department of Medicine, 10th Floor, 2775 Laurel Street, Vancouver, BC V5Z 1M9 Canada; 2 MTEK Sciences, 802-777 West Broadway, Vancouver, BC V5Z 1J5 Canada; 3 Department of Health Research Methods, Evidence, and Impact, McMaster University Medical Centre, 1280 Main Street West, 2C Area, Hamilton, ON L8S 4K1 Canada; 4 School of Population and Public Health, University of British Columbia, 2206 E Mall, Vancouver, BC V6T 1Z3 Canada; 5 Data and Methodology Program, CIHR Canadian HIV Trials Network, 588 – 1081 Burrard Street, Vancouver, BC V6Z 1Y6 Canada; 6 Knowledge Integration, Bill and Melinda Gates Foundation, 500 5th Ave N, Seattle, WA 98109 USA

**Keywords:** Master protocols, Basket trials, Umbrella trials, Platform trials, Multi-arm, multi-stage design

## Abstract

**Background:**

Master protocols, classified as basket trials, umbrella trials, and platform trials, are novel designs that investigate multiple hypotheses through concurrent sub-studies (e.g., multiple treatments or populations or that allow adding/removing arms during the trial), offering enhanced efficiency and a more ethical approach to trial evaluation. Despite the many advantages of these designs, they are infrequently used.

**Methods:**

We conducted a landscape analysis of master protocols using a systematic literature search to determine what trials have been conducted and proposed for an overall goal of improving the literacy in this emerging concept. On July 8, 2019, English-language studies were identified from MEDLINE, EMBASE, and CENTRAL databases and hand searches of published reviews and registries.

**Results:**

We identified 83 master protocols (49 basket, 18 umbrella, and 16 platform trials). The number of master protocols has increased rapidly over the last five years. Most have been conducted in the US (*n* = 44/83) and investigated experimental drugs (*n* = 82/83) in the field of oncology (*n* = 76/83). The majority of basket trials were exploratory (i.e., phase I/II; *n* = 47/49) and not randomized (*n* = 44/49), and more than half (*n* = 28/48) investigated only a single intervention. The median sample size of basket trials was 205 participants (interquartile range, Q3-Q1 [IQR]: 500–90 = 410), and the median study duration was 22.3 (IQR: 74.1–42.9 = 31.1) months. Similar to basket trials, most umbrella trials were exploratory (*n* = 16/18), but the use of randomization was more common (*n* = 8/18). The median sample size of umbrella trials was 346 participants (IQR: 565–252 = 313), and the median study duration was 60.9 (IQR: 81.3–46.9 = 34.4) months. The median number of interventions investigated in umbrella trials was 5 (IQR: 6–4 = 2). The majority of platform trials were randomized (*n* = 15/16), and phase III investigation (*n* = 7/15; one did not report information on phase) was more common in platform trials with four of them using seamless II/III design. The median sample size was 892 (IQR: 1835–255 = 1580), and the median study duration was 58.9 (IQR: 101.3–36.9 = 64.4) months.

**Conclusions:**

We anticipate that the number of master protocols will continue to increase at a rapid pace over the upcoming decades. More efforts to improve awareness and training are needed to apply these innovative trial design methods to fields outside of oncology.

**Electronic supplementary material:**

The online version of this article (10.1186/s13063-019-3664-1) contains supplementary material, which is available to authorized users.

## Background

Advancements in genomics, particularly in tumor sequencing, have improved our ability to differentiate cancers by their genetic mutations [1]. This has fueled the efforts towards “precision oncology”, in which therapies are selected to specifically target cancers on the basis of their genetic mutations. These innovative treatments are commonly referred to as targeted therapies [2]. However, it is unrealistic to investigate the broad spectrum of genetic sub-populations by conventional trial designs. Thus, “master protocol” frameworks have been proposed to provide a means of comprehensively and adaptively evaluating treatments from the field of oncology [3, 4].

The term “master protocol” refers to a single overarching design developed to evaluate multiple hypotheses, and the general goals are improving efficiency and establishing uniformity through standardization of procedures in the development and evaluation of different interventions [5, 6]. Under a common infrastructure, the master protocol may be differentiated into multiple parallel sub-studies to include standardized trial operational structures, patient recruitment and selection, data collection, analysis, and management [3–6].

Master protocols are often classified into “basket trials”, “umbrella trials”, and “platform trials” [3–6]. Basket trials refer to designs in which a targeted therapy is evaluated on multiple diseases that have common molecular alternations. Umbrella trials, on the other hand, evaluate multiple targeted therapies for a single disease that is stratified into subgroups by molecular alternation. Basket trials and umbrella trials employ a molecular screening protocol that allows either recruitment of different diseases with the common molecular alteration(s) or that differentiates the single disease into different molecular subtypes. Platform trials, also referred to as multi-arm, multi-stage (MAMS) design trials [7–10], are trials that evaluate several interventions against a common control group and can be perpetual [3, 5, 11, 12]. This design has pre-specified adaptation rules to allow dropping of ineffective intervention(s) and flexibility of adding new intervention(s) during the trial [3, 5, 11, 12].

Master protocols may be tailored and adapted to suit the research objectives of multiple clinical indications, but master protocols have not been well established in fields outside of oncology [4, 13]. There may be missed opportunities in research fields outside of oncology. Thus, improved understanding and awareness of these research designs are important for the research community. Methodological summaries of master protocols to date have not been comprehensive, and a cursory review of the literature returned no systematic literature reviews. With the intent of improving literacy in this emerging field, we conducted this comprehensive systematic literature review as a landscape analysis of master protocols.

## Methods

This systematic literature review was designed in accordance with the Preferred Reporting Items for Systematic Reviews and Meta-Analysis (PRISMA) guidelines [14] EQUATOR checklist for this review is provided in the Supplementary (Additional file 2).

### Data sources and searches

Systematic searches were conducted on July 8, 2019, in MEDLINE, EMBASE, and the Cochrane Central Register of Controlled Trials. As no validated literature search strategy has been published, our strategies were developed on the basis of a review of key papers, including the Draft Guidance of the US Food and Drug Administration (FDA) [3–6, 15]. We complemented the search terms of “master protocols”, “basket trials”, “umbrella trials”, and “platform trials” with several search terms specific to “adaptive trial designs” to improve the sensitivity of our search. The search strategies for each database are presented in Additional file 1: Tables S1–S3. We supplemented our database searches with a review of bibliographies from included publications. In addition, we searched trial registries (ClinicalTrials.gov and ISRCTN registry) for registered master protocols. Search terms used for ClinicalTrials.gov are reported in Additional file 1: Table S4. The list of published reviews related to master protocols that we reviewed is provided in Additional file 1: Table S5.

### Study inclusion and exclusion criteria

**Fig. 1:**
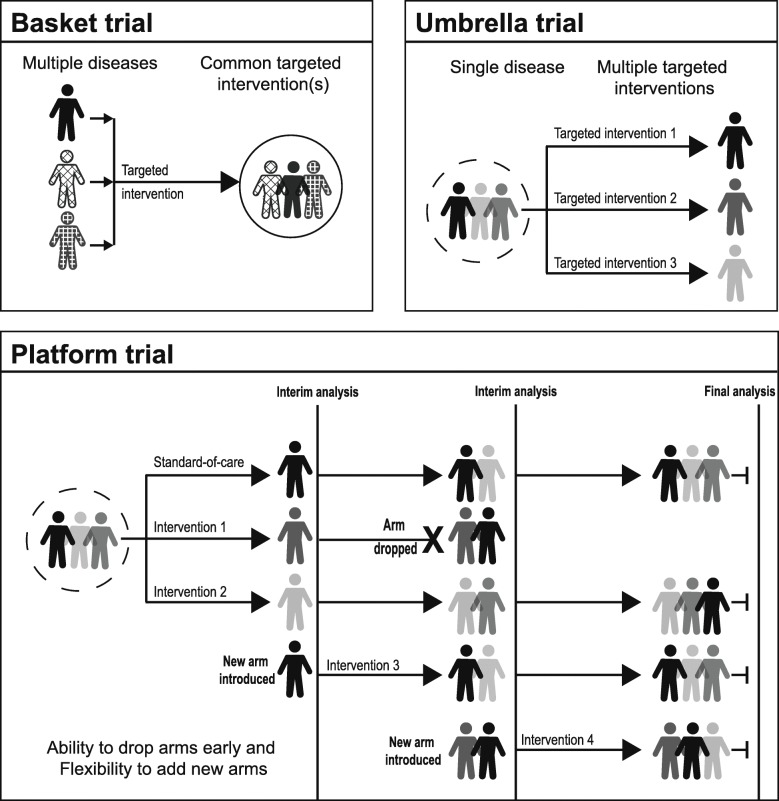
Graphical representation of basket trials, umbrella trials, and platform trials. This figure illustrates a simple graphical representation of basket, umbrella, and platform trials. There may be other forms of master protocols. The clip art in the figure was generated by the authors

Complete study eligibility is described in Table 1. In brief, we included peer-reviewed publications, conference abstracts, and clinical registry records reporting on master protocols (basket trials, umbrella trials, and platform trials) that have been proposed, are ongoing, or have already been conducted. We defined “basket trials” as any prospective clinical trials that investigated the utility (e.g., effectiveness, dosage, and safety) of intervention(s) in a study population of multiple diseases with common predictive biomarkers or other common predictive patient characteristics that can be used to predict whether a patient will respond to a specific intervention (or both) as the unifying eligibility criteria. We defined “umbrella trials” as any prospective clinical trials that investigated the utility of targeted interventions based on predictive biomarkers or other patient characteristics or both. In umbrella trials, the single disease population (e.g., single histology cancer) is stratified into multiple subgroups on predictive biomarkers or other characteristics or both. We defined “platform trials” as any clinical trials that allowed for the intervention arm(s) to be dropped and the flexibility of introducing new intervention(s) during the trial. Graphical displays of basket trials, umbrella trials, and platform trials are provided in Fig. 1. We excluded non-English language studies.

**Table 1:** PICOS (population, intervention, comparator, outcomes, study design) criteria

Category	Inclusion criteria
Population	Humans
Interventions	No restrictions
Comparator	No restrictions
Outcomes	No restrictions
Study design	Master protocols were defined as a single overarching protocol that has been designed to be divided into multiple sub-studies that could allow for evaluation of multiple interventional hypotheses. These included: - Basket trials - Umbrella trials - Platform trials
Other	Peer-reviewed publications and conference abstracts with results or published protocols in the English language

‘Basket trials’ were defined as any prospective clinical trials that tested the utility (e.g., effectiveness, dosage, and safety) of intervention(s) in a study population of multiple diseases with common predictive biomarkers and/or other common predictive patient characteristics that can be used to predict whether a patient will respond to a specific intervention as the unifying eligibility criteria

‘Umbrella trials’ were defined as any prospective clinical trials that tested the utility of targeted interventions based on predictive biomarkers or other patient characteristics or both; in umbrella trials, the single disease study population is stratified into multiple subgroups on predictive biomarkers or other characteristics or both

‘Platform trials’ were defined as any clinical trials that allowed the intervention arm(s) to be dropped and the flexibility of introducing new intervention(s) during the trial. Platform trials are sometimes referred to as multi-arm, multi-stage (MAMS) designs, but the MAMS designs that do not allow flexibility of adding new arms during the trial are not truly platform trials

Two reviewers (JJHP and MJZ) independently reviewed all abstracts and proceedings identified in the literature searches. The full-text publications of potentially relevant abstracts were then retrieved and assessed for eligibility. Two reviewers also screened the bibliographies of published literature reviews on master protocols (JJHP and ES) and trial registries (JJHP and LD). Discrepancies in study selection were resolved by discussion or, when necessary, by a third investigator (KT or EJM).

### Data extraction

Study design elements, patient characteristics, and outcomes were extracted independently by two investigators (JJHP and ES) using a standardized, piloted data extraction form. We recorded information on trial registry, trial recruitment status, phase, randomization, masking, number of clinical centers, sample size, trial duration, interventions and control, disease area, age of population, number of conventional diseases recruited, key eligibility for stratification, number of subgroups defined, and geographic location of the master protocols. Discrepancies were resolved by discussion.

### Data synthesis

A meta-analysis was not conducted for this study, and we present the findings of this landscape analysis descriptively. We report on temporal trends of master protocols, geographical representation, and trial and disease characteristics of each of the three master protocols (basket trials, umbrella trials, and platform trials).

### Role of the funding source

This study was not funded.

## Results

### Literature search

The study selection process is presented in Additional file 1: Figure S1. We identified 5869 abstracts from our database searches, and 140 more records were identified through hand searches of bibliographies and trial registries. Of these, 639 records were selected for full-text review. In total, 214 publications describing 83 trials met our inclusion criteria. Thirty-four trials were available only through trial registries, and three trials were in the pre-recruitment phase (NCT03339843, NCT03915678, and NCT03872427). A complete list of trials and the corresponding citations is provided in Additional file 1: Tables S6–S8. In summary, we identified 49 basket trials, 18 umbrella trials, and 16 platform trials.

### Trends of master protocols

**Fig. 2:**
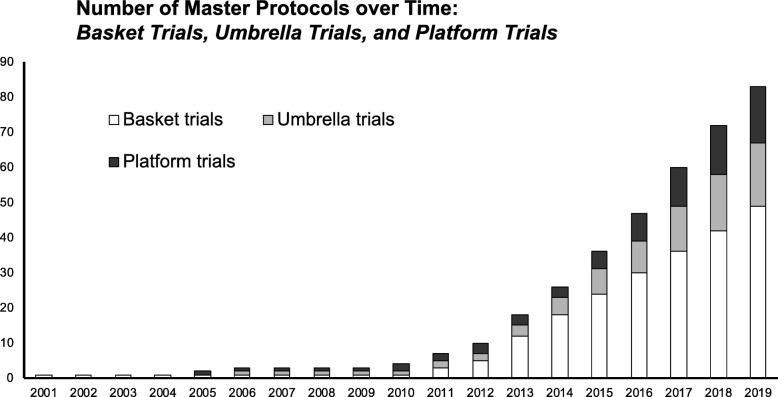
Trends of master protocols over time. This figure illustrates the accumulating number of basket (white), umbrella (gray), and platform (black) trials over time. The clip art in the figure was generated by the authors

There has been a rapid increase in the number of master protocols published in the last five years (Fig. 2). From our literature search, we identified nine completed and published master protocol trials, including results. The first master protocol conducted was a basket trial called the Imatinib Target Exploration Consortium Study B2225 [16, 17], which started in 2001. This was followed by the platform trial STAMPEDE, which was first proposed in 2005 [8, 9, 18–28]. We identified 68 ongoing master protocols (39 basket trials, 17 umbrella trials, and 12 platform trials) recruiting patients; of these, 11 basket trials [29–38], eight umbrella trials [39–46], and four platform trials [24, 47–50] have published results (Additional file 1: Table S10).

At the time of writing (August 1, 2019), one platform trial (LEAP; NCT03092674) is suspended for an unscheduled safety data review [51]. EBOLA (NCT02380 625), a platform trial supported by the Bill & Melinda Gates Foundation in response to the 2014 West Africa Ebola outbreak, has been terminated, as it could not be launched in time in response to the outbreak [52].

### Trial characteristics of master protocols

**Fig. 3:**
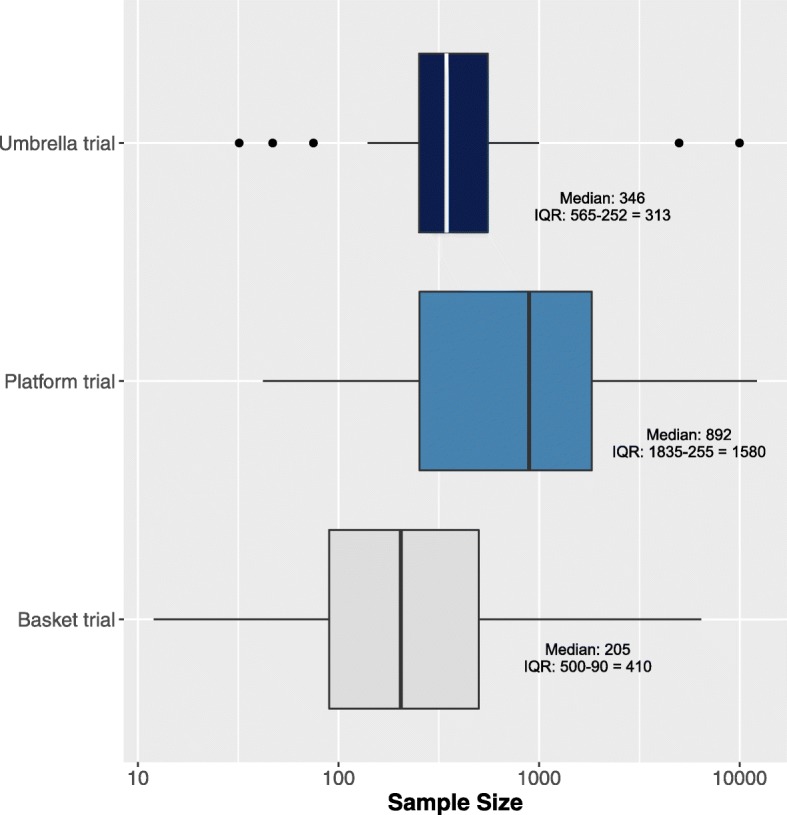
Sample size distribution of master protocols. *Abbreviation*: *IQR* interquartile range. The clip art in the figure was generated by the authors

Trial characteristics of the master protocols are presented in Additional file 1: Table S9, and the sample size distribution of these master protocols displayed as box plots is provided in Fig. 3.

The majority of master protocols were basket designs, and 49 are identified in the current review. Among basket trials, all but one involved a drug investigation (*n* = 48/49); NCT03003195 was the exception as a proposed vaccine basket trial. The majority of basket trials were exploratory (i.e., phase I or II; *n* = 47/49) and were open-label (*n* = 46/49); more than half of the included basket trials investigated only a single intervention arm (*n* = 28/48; one did not report information on the number of interventions), and the majority did not involve a control group or randomization (*n* = 44/49). The median sample size of basket trials was 205 participants (interquartile range, Q3-Q1 [IQR]: 500–90 = 410), and the median study duration was 22.3 (IQR: 74.1–42.9 = 31.1) months. ALCHEMIST (NCT02193282, NCT02595944, and NCT02201992) and CLUSTER (NCT02059291) [53–55] were the only phase III basket trials, which were comprised of three interventions arms and were of an open-label design.

Eighteen umbrella trials were identified. All umbrella trials investigated experimental drugs, and eight out of the 18 trials used randomization to assign patients to different arms. The median sample size of umbrella trials was 346 participants (IQR: 565–252 = 313), and the median study duration was 60.9 (IQR: 81.3–46.9 = 34.4) months. The median number of interventions investigated in umbrella trials was 5 (IQR: 6–4 = 2). Similar to basket trials, the majority of umbrella trials were exploratory (*n* = 16/18) and open-label (n = 16/17; one did not report information on blinding).

Our review returned 16 platform trials. All of the platform trials involved investigation of experimental drugs. The median sample size was 892 (IQR: 1835–255 = 1580), and the median study duration was 58.9 (IQR: 101.3–36.9 = 64.4) months. Nearly all platform trials were of open-label design (*n* = 12/14; two trials did not report information on blinding), similar to basket and umbrella trials. However, phase III investigation was more common among platform trials (*n* = 7/15; one did not report information on phase) in contrast to basket and umbrella trials; four of these seven platform trials were seamless II/III trials. In the majority of platform trials, patients were assigned by randomization (*n* = 15/16). PRISM (NCT03527147) was the only non-randomized platform trial, although this is currently a phase I study. However, the trial registry of PRISM indicates that future arms may be added. In STAMPEDE [8, 9, 18–28] and I-SPY2 [49, 50, 56–61], several agents have graduated from the phase II evaluation with seamless transitions into phase III evaluations. The phase III evaluation for the I-SPY program is called I-SPY3.

### Disease characteristics of master protocols

The patient and disease characteristics of master protocols are provided in Additional file 1: Table S10. Most studies were in adult populations (*n* = 69/83), and nearly all were in the field of oncology (*n* = 76/83). No umbrella trials were conducted outside of oncology. Notably, two basket trials were conducted for other clinical indications, namely hereditary periodic fevers (CLUSTER; NCT02059291) [53–55] and complement-mediated disorders (TNT0009 Basket trial). Additionally, five platform trials have been designed for influenza (ALIC4E; ISRCTN27908921) [62], Ebola (EBOLA) [52], pneumonia (REMAP-CAP; NCT02735707), pre-operative surgery (UPMC REMAP; NCT03861767), and Alzheimer’s disease (The DIAN-TU platform; NCT01760005) [48].

### Geographic representation of master protocols

**Fig. 4:**
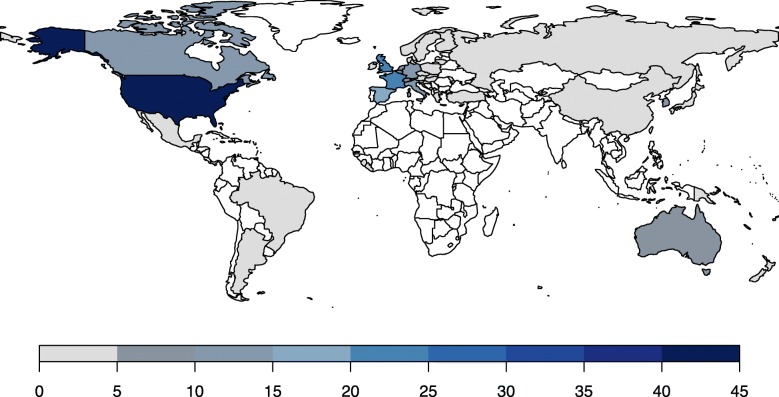
Geographical representation of master protocols. This figure illustrates the accumulating number of basket (white), umbrella (gray), and platform (black) trials over time. The clip art in the figure was generated by the authors

The information on the geographical representation of the current master protocols is provided in Additional file 1: Table S11. The majority of current master protocols have taken place in the US (*n* = 44/83) (Fig. 4). Other high-income countries such as the UK (*n* = 25), France (*n* = 23), Spain (*n* = 17), and Canada (*n* = 13) were the next most common countries. There were no master protocols observed from low-income countries, although the EBOLA (NCT02380625) trial had been proposed for Guinea, Sierra Leone, and Liberia [52]. Two upper-middle-income countries, Brazil and Mexico, were involved in the DIAN-TU platform trial (NCT01760005), but these countries accounted for only three of 36 study sites [48]. China, an upper-middle-income country, has centers participating in FUTURE (NCT03805399), GBM AGILE [47, 56, 63], TRUMP (NCT03574402), and VE-BASKET (NCT01524978) [64, 65] trials, but it should be noted that China accounts for only a minority of study sites in GBM AGILE and VE-BASKET.

## Discussion

To the best of our knowledge, this is the first landscape analysis of master protocols. This was achieved through a methodologically robust and rigorous systematic literature review that included queries of medical literature databases, reference lists of included studies, and clinical trial registries. Unlike previous publications on master protocols that were limited in scope to select only specific studies, this review catalogues all master protocols that have been conducted or proposed to date. Of the 83 master protocols (49 basket trials, 18 umbrella trials, and 16 platform trials), the majority have involved investigation of experimental drugs in adult patients for the field of oncology.

Our study may have been limited by variability of terminology and lack of standardized nomenclatures and indexing of master protocols in the medical databases. However, we believe that this was offset by our rigorous approach that had strong supplemental searching strategies inclusive of several search terms on adaptive trial designs. We first reviewed the key papers on master protocols to gain an overview of the existing literature [3–6, 15] before coming up with our search strategy. (We recommend that readers review these key publications.) Then developed search terms were complemented by hand searches of bibliographies of 52 published reviews that we found before and during the screening process (Additional file 1: Table S5) and international trial registries.

We have identified several directions for future research. An improved approach to standardized nomenclature and database indexing is essential to improve the identification and retrieval of these study designs. Moreover, efforts are needed to improve the awareness and technical expertise [3–6, 15] of master protocols to investigators in fields outside of oncology and in geographic regions outside of high-income countries (e.g., the US). Platform trials, by nature, are potentially perpetual and permit research questions to evolve over time in the context of new information [11, 12]. Basket trials and umbrella trials have had considerable emphasis and dependencies on the accuracy of genomic biomarkers used to characterize cancers, in addition to their histology and location [5], but it is important to point out that other baseline patient characteristics may be used to determine the intervention strategies. Thus, an emphasis on the study of how genomic screening tests impact the operational characteristics of these biomarker trials is warranted. Comparing different nomenclatures used in published trials and reviews may also be warranted in order to come up with a consensus on master protocols.

## Conclusion

This is the first systematic review-based bibliometric analysis of master protocols. The number of master protocols, especially in the last five years, has increased dramatically and we anticipate that this trend will continue over the coming years. Master protocols, particularly platform trials, have the potential to improve the efficiency across the broad spectrum of clinical trial research. This study was carried out at an opportunistic time, as the FDA released draft guidance on master protocols in September 2018 [15]. We anticipate that this landscape analysis may be useful for regulatory agencies as well as clinical investigators and readers who are looking to broaden their expertise in this emerging field.

## Additional files

Additional file 1: Supplementary Appendix. Supplementary to “Systematic review of basket trials, umbrella trials, and platform trials: A landscape analysis of master protocols”. (DOCX 604 kb)

Additional file 2: EQUATOR Checklist. EQUATOR Checklist for “Systematic review of basket trials, umbrella trials, and platform trials: A landscape analysis of master protocols”. (DOC 63 kb)

## Data Availability

All data generated or analyzed during this study are included in this published article.

## Abbreviations

FDA: US Food and Drug Administration
IQR: Interquartile range
