# Supplementary material for: Systematic review of basket trials, umbrella trials, and platform trials: a landscape analysis of master protocols
Source: Trials. 2019 Sep 18;20:572. doi: 10.1186/s13063-019-3664-1 (PMC6751792; doi:10.1186/s13063-019-3664-1)
Supplement: Supplementary file 1 — Supplementary Appendix. Supplementary to “Systematic review of basket trials, umbrella trials, and platform trials: A landscape analysis of master protocols”. (DOCX 604 kb) [file 13063_2019_3664_MOESM1_ESM.docx]

Supplementary appendix

**Supplementary to “***Systematic review of basket trials, umbrella trials, and platform trials: A landscape analysis of master protocols”*

**Table of Contents**

[Supplementary appendix 1](#_Toc535399625)

[Appendix 1. Literature search strategy and existing reviews 4](#_Toc535399626)

[Table S1. Search strategy Medline 4](#_Toc535399627)

[Table S2. Search strategy for EMBASE 5](#_Toc535399628)

[Table S3. Search strategy for CENTRAL 6](#_Toc535399629)

[Table S4. Search strategy for ClinicalTrials.gov and ISRCTN registry 7](#_Toc535399630)

[Table S5. Existing reviews and guidance on master protocols 8](#_Toc535399631)

[Appendix 2. Study selection 10](#_Toc535399632)

[Figure S1. Study flow chart 10](#_Toc535399633)

[Appendix 3. List of included studies 11](#_Toc535399634)

[Table S6. List of included studies: Basket trials 11](#_Toc535399635)

[Table S7. List of included studies: Umbrella trials 14](#_Toc535399636)

[Table S8. List of included studies: Platform trials 16](#_Toc535399637)

[Appendix 4. Trial and disease characteristics 18](#_Toc535399638)

[Table S9. Trial characteristics of master protocols 18](#_Toc535399639)

[Table S10. Disease characteristics of master protocols 20](#_Toc535399640)

[Reference: 22](#_Toc535399641)

1. Literature search strategy and existing reviews

Table S1. Search strategy Medline

Executed on July 8, 2019

| **No.** | **Terms** | **Hits** | **Comments** |
| --- | --- | --- | --- |
| 1 | (Master protocol or master trial protocol or trial master protocol).mp. | 69 | Master protocol |
| 2 | (Platform trial or platform clinical trial or adaptive platform trial or platform adaptive trial).mp. | 42 | Platform trial design |
| 3 | (Umbrella trial or umbrella clinical trial or adaptive umbrella trial or umbrella adaptive trial).mp. | 11 | Umbrella trial |
| 4 | (Basket trial or basket clinical trial or adaptive basket trial or basket adaptive trial).mp. | 48 | Basket trial |
| 5 | (Multi-arm multi-stage or MAMS).mp. | 297 | Multi-arm multi-stage design |
| 6 | ((Multi-stage or multi stage or multistage) and (clinical trial or randomized trial or randomised trial)).mp. | 298 |  |
| 7 | Adaptive clinical trials.mp. | 108 | General terms for adaptive trial designs |
| 8 | Adaptive clinical trial.mp. | 98 |  |
| 9 | Adaptive design.mp. | 675 |  |
| 10 | Flexible trial design.mp. | 2 |  |
| 11 | (Bayesian clinical trial or bayesian adaptive).mp. | 294 |  |
| 12 | Adaptive group sequential design.mp. | 13 | Adaptive group sequential design |
| 13 | Group sequential design.mp. | 150 |  |
| 14 | (Interim monitoring or stochastic curtailment or futility analysis).mp. | 256 | Interim monitoring and stochastic curtailment |
| 15 | (Predictive probabilit* or conditional power or bayesian predictive probabilit*).mp. | 484 |  |
| 16 | ((Predictive power or posterior probabilit*) and (interim monitoring or interim analys*)).mp. | 30 |  |
| 17 | ((Sample size or sample-size) adj3 (reestimation or re-estimation or re estimation)).mp. | 144 | Sample size re-estimation |
| 18 | ((Sample size or sample-size) adj3 (reassessment or re-assessment or re assessment)).mp. | 47 |  |
| 19 | ((Sample size or sample-size) adj3 adjustment).mp. | 103 |  |
| 20 | ((Sample size or sample-size) adj3 modification).mp. | 24 |  |
| 21 | (Enrichment design or population enrichment or population enrichment design or bayesian adaptive enrichment).mp. | 111 | Population enrichment design or biomarker enrichment design |
| 22 | (biomarker design or biomarker adaptive or biomarker adaptive design or biomarker adjusted).mp. | 30 |  |
| 23 | (Responsive adaptive randomization or responsive adaptive randomisation or responsive adaptive allocation or adaptive allocation or adaptive randomization or adaptive randomisation or response adaptive).mp. | 389 | Responsive adaptive randomization |
| 24 | (Play the winner or play-the-winner or pick the winner or pick-the-winner or drop the loser or drop-the-loser).mp. | 79 |  |
| 25 | (Adaptive dose rang* or adaptive dose or bayesian dose).mp. | 265 |  |
| 26 | (Seamless design or adaptive seamless or operationally seamless or operational seamless or inferentially seamless or inferential seamless).mp. | 49 | Seamless design |
| 27 | ("Seamless 2-3" or "seamless 2 to 3" or "seamless 2/3" or "seamless phase ii/iii" or "seamless phase 2/3").mp. | 48 |  |
| 28 | ("Seamless 1-2" or "seamless 1 to 2" or "seamless 1/2" or "seamless phase i/ii" or "seamless phase 1/2").mp. | 17 |  |
| 29 | or/1-28 | 3339 |  |
| 30 | Humans/ | 17835321 |  |
| 31 | Animals/ | 6430856 |  |
| 32 | (29 and 30) not 31 | 2288 | Limiting to studies on humans |
| 33 | limit 32 to english language | 2232 | English language |


Table S2. Search strategy for EMBASE

Executed on July 8, 2019

| **No.** | **Terms** | **Hits** | **Comments** |
| --- | --- | --- | --- |
| 1 | (Master protocol or master trial protocol or trial master protocol).mp. | 126 | Master protocol |
| 2 | (Platform trial or platform clinical trial or adaptive platform trial or platform adaptive trial).mp. | 75 | Platform trial design |
| 3 | (Umbrella trial or umbrella clinical trial or adaptive umbrella trial or umbrella adaptive trial).mp. | 56 | Umbrella trial |
| 4 | (Basket trial or basket clinical trial or adaptive basket trial or basket adaptive trial).mp. | 134 | Basket trial |
| 5 | (Multi-arm multi-stage or MAMS).mp. | 436 | Multi-arm multi-stage design |
| 6 | ((Multi-stage or multi stage or multistage) and (clinical trial or randomized trial or randomised trial)).mp. | 695 |  |
| 7 | Adaptive clinical trials.mp. | 121 | General terms for adaptive trial designs |
| 8 | Adaptive clinical trial.mp. | 224 |  |
| 9 | Adaptive design.mp. | 1186 |  |
| 10 | Flexible trial design.mp. | 6 |  |
| 11 | (Bayesian clinical trial or bayesian adaptive).mp. | 401 |  |
| 12 | Adaptive group sequential design.mp. | 17 | Adaptive group sequential design |
| 13 | Group sequential design.mp. | 243 |  |
| 14 | (Interim monitoring or stochastic curtailment or futility analysis).mp. | 515 | Interim monitoring and stochastic curtailment |
| 15 | (Predictive probabilit* or conditional power or bayesian predictive probabilit*).mp. | 773 |  |
| 16 | ((Predictive power or posterior probabilit*) and (interim monitoring or interim analys*)).mp. | 70 |  |
| 17 | ((Sample size or sample-size) adj3 (reestimation or re-estimation or re estimation)).mp. | 233 | Sample size re-estimation |
| 18 | ((Sample size or sample-size) adj3 (reassessment or re-assessment or re assessment)).mp. | 66 |  |
| 19 | ((Sample size or sample-size) adj3 adjustment).mp. | 150 |  |
| 20 | ((Sample size or sample-size) adj3 modification).mp. | 39 |  |
| 21 | (Enrichment design or population enrichment or population enrichment design or bayesian adaptive enrichment).mp. | 202 | Population enrichment design or biomarker enrichment design |
| 22 | (biomarker design or biomarker adaptive or biomarker adaptive design or biomarker adjusted).mp. | 80 |  |
| 23 | (Responsive adaptive randomization or responsive adaptive randomisation or responsive adaptive allocation or adaptive allocation or adaptive randomization or adaptive randomisation or response adaptive).mp. | 528 | Responsive adaptive randomization |
| 24 | (Play the winner or play-the-winner or pick the winner or pick-the-winner or drop the loser or drop-the-loser).mp. | 144 |  |
| 25 | (Adaptive dose rang* or adaptive dose or bayesian dose).mp. | 393 |  |
| 26 | (Seamless design or adaptive seamless or operationally seamless or operational seamless or inferentially seamless or inferential seamless).mp. | 89 | Seamless design |
| 27 | ("Seamless 2-3" or "seamless 2 to 3" or "seamless 2/3" or "seamless phase ii/iii" or "seamless phase 2/3").mp. | 81 |  |
| 28 | ("Seamless 1-2" or "seamless 1 to 2" or "seamless 1/2" or "seamless phase i/ii" or "seamless phase 1/2").mp. | 20 |  |
| 29 | or/1-28 | 5648 |  |
| 30 | Humans/ | 11276274 |  |
| 31 | Animals/ | 1220087 |  |
| 32 | (29 and 30) not 31 | 1401 | Limiting to studies on humans |
| 33 | limit 32 to english language | 1343 | English language |

Table S3. Search strategy for CENTRAL

Executed on July 8, 2019

| **No.** | **Terms** | **Hits** | **Comments** |
| --- | --- | --- | --- |
| 1 | (Master protocol or master trial protocol or trial master protocol).mp. | 32 | Master protocol |
| 2 | (Platform trial or platform clinical trial or adaptive platform trial or platform adaptive trial).mp. | 48 | Platform trial design |
| 3 | (Umbrella trial or umbrella clinical trial or adaptive umbrella trial or umbrella adaptive trial).mp. | 21 | Umbrella trial |
| 4 | (Basket trial or basket clinical trial or adaptive basket trial or basket adaptive trial).mp. | 26 | Basket trial |
| 5 | (Multi-arm multi-stage or MAMS).mp. | 57 | Multi-arm multi-stage design |
| 6 | ((Multi-stage or multi stage or multistage) and (clinical trial or randomized trial or randomised trial)).mp. | 210 |  |
| 7 | Adaptive clinical trials.mp. | 7 | General terms for adaptive trial designs |
| 8 | Adaptive clinical trial.mp. | 57 |  |
| 9 | Adaptive design.mp. | 399 |  |
| 10 | Flexible trial design.mp. | 4 |  |
| 11 | (Bayesian clinical trial or bayesian adaptive).mp. | 100 |  |
| 12 | Adaptive group sequential design.mp. | 7 | Adaptive group sequential design |
| 13 | Group sequential design.mp. | 77 |  |
| 14 | (Interim monitoring or stochastic curtailment or futility analysis).mp. | 308 | Interim monitoring and stochastic curtailment |
| 15 | (Predictive probabilit* or conditional power or bayesian predictive probabilit*).mp. | 152 |  |
| 16 | ((Predictive power or posterior probabilit*) and (interim monitoring or interim analys*)).mp. | 21 |  |
| 17 | ((Sample size or sample-size) adj3 (reestimation or re-estimation or re estimation)).mp. | 50 | Sample size re-estimation |
| 18 | ((Sample size or sample-size) adj3 (reassessment or re-assessment or re assessment)).mp. | 18 |  |
| 19 | ((Sample size or sample-size) adj3 adjustment).mp. | 31 |  |
| 20 | ((Sample size or sample-size) adj3 modification).mp. | 6 |  |
| 21 | (Enrichment design or population enrichment or population enrichment design or bayesian adaptive enrichment).mp. | 54 | Population enrichment design or biomarker enrichment design |
| 22 | (biomarker design or biomarker adaptive or biomarker adaptive design or biomarker adjusted).mp. | 6 |  |
| 23 | (Responsive adaptive randomization or responsive adaptive randomisation or responsive adaptive allocation or adaptive allocation or adaptive randomization or adaptive randomisation or response adaptive).mp. | 256 | Responsive adaptive randomization |
| 24 | (Play the winner or play-the-winner or pick the winner or pick-the-winner or drop the loser or drop-the-loser).mp. | 86 |  |
| 25 | (Adaptive dose rang* or adaptive dose or bayesian dose).mp. | 104 |  |
| 26 | (Seamless design or adaptive seamless or operationally seamless or operational seamless or inferentially seamless or inferential seamless).mp. | 38 | Seamless design |
| 27 | ("Seamless 2-3" or "seamless 2 to 3" or "seamless 2/3" or "seamless phase ii/iii" or "seamless phase 2/3").mp. | 28 |  |
| 28 | ("Seamless 1-2" or "seamless 1 to 2" or "seamless 1/2" or "seamless phase i/ii" or "seamless phase 1/2").mp. | 8 |  |
| 29 | or/1-28 | 1847 |  |
| 30 | Humans/ | 561312 |  |
| 31 | Animals/ | 9671 |  |
| 32 | (29 and 30) not 31 | 361 | Limiting to studies on humans |
| 33 | limit 32 to english language | 361 | English language |

Table S4. Search strategy for ClinicalTrials.gov and ISRCTN registry

Executed on July 13^th^, 2019

| **Search details** | **URL** |
| --- | --- |
| **Clinicaltrials.gov** | |
| "Master protocol" | <https://clinicaltrials.gov/ct2/results?cond=&term=%22master+protocol%22&cntry=&state=&city=&dist=&Search=Search> |
| "Basket trial" | <https://clinicaltrials.gov/ct2/results?cond=&term=%22basket+trial%22&cntry=&state=&city=&dist=&Search=Search> |
| "Umbrella trial" | <https://clinicaltrials.gov/ct2/results?cond=&term=%22Umbrella+trial%22&cntry=&state=&city=&dist=&Search=Search> |
| "Platform trial" | <https://clinicaltrials.gov/ct2/results?cond=&term=%22platform+trial%22&cntry=&state=&city=&dist=&Search=Search> |
| Multi-arm multi-stage | <https://clinicaltrials.gov/ct2/results?cond=&term=Multi-arm+multi-stage&cntry=&state=&city=&dist=&Search=Search> |
| **ISRCTN registry** | |
| Master protocol | http://www.isrctn.com/search?q=master+protocol |
| Basket trial | http://www.isrctn.com/search?q=Basket+trial |
| Umbrella trial | <http://www.isrctn.com/search?q=umbrella+trial> |
| “Platform trial” | http://www.isrctn.com/search?q=%22platform+trial%22 |
| Multi-arm multi-stage | http://www.isrctn.com/search?q=Multi-arm+multi-stage |

Table S5. Existing reviews and guidance on master protocols

| **Study ID** | **Title** | **Details** |
| --- | --- | --- |
| Abrams 2014[1] | National cancer institute's precision medicine initiatives for the new national clinical trials network | Master protocol |
| Bando 2016[2] | Perspectives on research activity in the USA on cancer precision medicine | Master protocol |
| Beckman 2016[3] | Adaptive design for a confirmatory basket trial in multiple tumor types based on a putative predictive biomarker | Basket trials |
| Berry 2015[4] | The brave new world of clinical cancer research: Adaptive biomarker-driven trials integrating clinical practice with clinical research | Master protocol: Basket trial; Platform trial |
| Berry 2015[5] | The platform trial: An efficient strategy for evaluating multiple treatments | Master protocol: Platform |
| Blumenthal 2018[6] | Current Status and Future Perspectives on Neoadjuvant Therapy in Lung Cancer | Umbrella trials |
| Bratton 2013[7] | A multi-arm multi-stage clinical trial design for binary outcomes with application to tuberculosis | Master protocol: MAMS |
| Butler 2018[8] | Answering patient-centred questions efficiently: Response-adaptive platform trials in primary care | Master protocol: Platform |
| Chen 2018[9] | Basket trial of trk inhibitors demonstrates efficacy in trk fusion-positive cancers | Basket trials |
| Collignon 2018[10] | Adaptive designs in clinical trials: From scientific advice to marketing authorisation to the european medicine agency | Other (EMA) |
| Cunanan 2017[11] | An efficient basket trial design | Basket trials |
| FDA 2018[12] | Master Protocols: Efficient Clinical Trial Design Strategies to Expedite Development of Oncology Drugs and Biologics Guidance for Industry (Draft Guidance) | Master protocols |
| FDA 2018[13] | Adaptive Designs for Clinical Trials of Drugs and Biologics Guidance for Industry (Draft Guidance) | Platform trials (and adaptive designs) |
| Fountzilas 2018[14] | Overview of precision oncology trials: Challenges and opportunities | Master protocols |
| Grignolo 2018[15] | Improving drug development and patient access with the right people, processes, and culture: What needs to happen right now to bring better medicines to the patients who need them | Master protocols |
| Heckman-Stoddard 2014[16] | Precision medicine clinical trials: Defining new treatment strategies | Master protocols |
| Herbst 2015[17] | Lung Master Protocol (Lung-MAP)—A Biomarker-Driven Protocol for Accelerating Development of Therapies for Squamous Cell Lung Cancer: SWOG S1400 | Umbrella trials |
| Hirakawaa 2018[18] | Master protocol trials in oncology: Review and new trial designs | Master protocol: All |
| Hobbs 2018[19] | Bayesian basket trial design with exchangeability monitoring | Basket trials |
| Hobbs 2018[20] | Controlled multi-arm platform design using predictive probability | Master protocol: Platform |
| Iams 2018[21] | stop fReTting the Target: next-Generation ReT Inhibitors Have Arrived | Master protocols |
| Khan 2018[22] | Accelerating pediatric cancer drug development: Challenges and opportunities for pediatric master protocols | Master protocols |
| Kourie 2016[23] | Why balls are not put inside the basket? A reflection on testicular cancer clinical trial design | Basket trials |
| Lam 2018[24] | Master protocols in lung cancer: Experience from lung master protocol | Master protocols |
| Ledford 2013[25] | 'Master protocol' aims to revamp cancer trials | Master protocols |
| Lee 2011[26] | Biomarker-based bayesian adaptive designs for targeted agent development | Master protocol |
| Lih 2017[27] | Considerations of developing an ngs assay for clinical applications in precision oncology: The nci-match ngs assay experience | Basket trials |
| Lin 2017[28] | Comparison of multi-arm multi-stage design and adaptive randomization in platform clinical trials | Master protocol: Platform |
| Manca 2017[29] | Leading Antibacterial Laboratory Research by Integrating Conventional and Innovative Approaches: The Laboratory Center of the Antibacterial Resistance Leadership Group | Master protocol |
| Mandrekar 2015[30] | Improving clinical trial efficiency: Thinking outside the box | Master protocol: Umbrella |
| Martin 2017[31] | Using trial infrastructure efficiently: Platform, factorial, and umbrella trial designs | Master protocol: All |
| McCabe 2017[32] | Novel developments in paediatric cancer | Basket trials |
| Mullard 2015[33] | Nci-match trial pushes cancer umbrella trial paradigm | Master protocol: Umbrella |
| Ornes 2016[34] | Core concept: Basket trial approach capitalizes on the molecular mechanisms of tumors | Basket trials |
| Parmar 2017[35] | Testing many treatments within a single protocol over 10years at mrc clinical trials unit at ucl: Multi-arm, multi-stage platform, umbrella and basket protocols | Master protocol: All |
| Phillips 2012[36] | Innovative trial designs are practical solutions for improving the treatment of tuberculosis | Master protocol: MAMS |
| Pitts 2018[37] | 21st-century oncology drug safety via new-age regulatory standards and practices | Master protocols |
| Redman 2015[38] | The master protocol concept | Master protocols |
| Renfro 2017[39] | Statistical controversies in clinical research: basket trials, umbrella trials, and other master protocols: a review and examples | Master protocol: All |
| Saville 2016[40] | Efficiencies of platform clinical trials: A vision of the future | Master protocol: Platform |
| Simon 2016[41] | Genomic alteration-driven clinical trial designs in oncology | Master protocol |
| Steuer 2015[42] | Innovative clinical trials: The lung-map study | Umbrella trials |
| Szczykutowicz 2015[43] | Ct protocol management: Simplifying the process by using a master protocol concept | Master protocol |
| Tanguturi 2017[44] | Leveraging molecular datasets for biomarker-based clinical trial design in glioblastoma | Master protocol |
| Tao 2018[45] | Basket studies: Redefining clinical trials in the era of genome-driven oncology | Basket trials |
| Teo 2018[46] | Drug development for noncastrate prostate cancer in a changed therapeutic landscape | Master protocols |
| Tolles 2018[47] | Adaptive and platform trials in remote damage control resuscitation | Platform trials |
| Trusheim 2016[48] | Pipelines: Creating comparable clinical knowledge efficiently by linking trial platforms | Master protocol: Platform |
| Ventz 2017[49] | Bayesian response-adaptive designs for basket trials | Basket trials |
| Walter 2017[50] | Intergroup leap trial (s1612): A randomized phase 2/3 platform trial to test novel therapeutics in medically less fit older adults with acute myeloid leukemia | Master protocol: Platform |
| Woodcock 2017[51] | Master Protocols to Study Multiple Therapies, Multiple Diseases, or Both | Master protocol: All |
| Zardavas 2015[52] | Clinical trials of precision medicine through molecular profiling: Focus on breast cancer | Master protocols and adaptive trial designs |

1. Study selection

Figure S1. Study flow chart


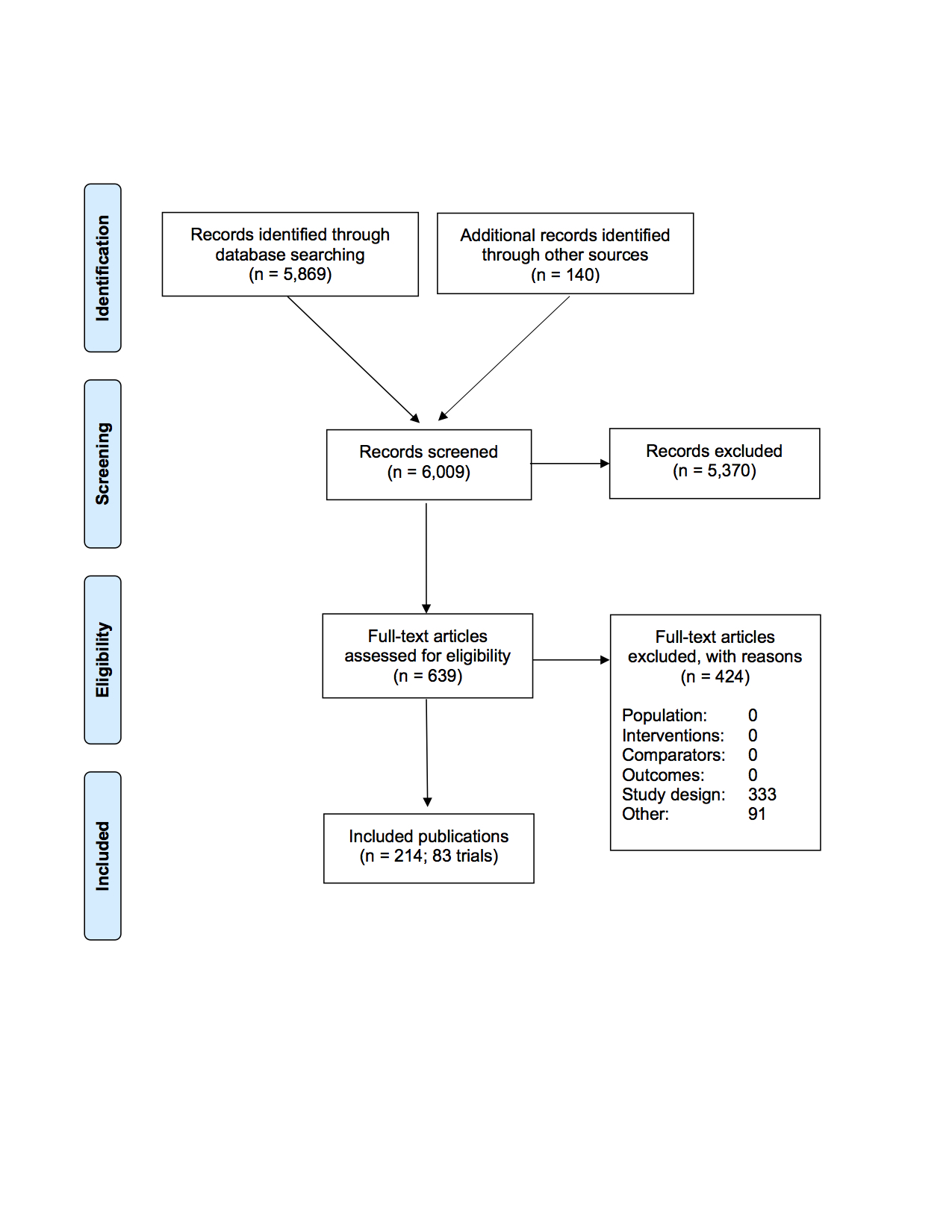


1. List of included studies

Table S6. List of included studies: Basket trials

| **Trial ID** | **Study ID** | **Year** | **Title** |
| --- | --- | --- | --- |
| **Basket trials** | | | |
| AcSe´ | NCT02304809 | 2014 | Phase 2 Study Assessing Secured Access to Vemurafenib for Patients With Tumors Harboring BRAF Genomic Alterations (AcSé) |
| Ado-trastuzumab Basket trial | Li 2017 | 2017 | Ado-trastuzumab emtansine in patients with her2 mutant lung cancers: Results from a phase ii basket trial |
| Ado-trastuzumab Basket trial | Li 2018 | 2018 | A multi-histology basket trial of adotrastuzumab emtansine in patients with her2 amplified cancers |
| Ado-trastuzumab Basket trial | NCT02675829 | 2016 | Trial of Ado-Trastuzumab Emtansine for Patients With HER2 Amplified or Mutant Cancers |
| AGADIR | NCT03915678 | 2019 | Atezolizumab Combined With Intratumoral G100 AnD Immunogenic Radiotherapy in Patients With Advanced Solid Tumors (AGADIR) |
| ALCHEMIST | NCT02193282 | 2014 | Erlotinib Hydrochloride in Treating Patients With Stage IB-IIIA Non-small Cell Lung Cancer That Has Been Completely Removed by Surgery (An ALCHEMIST Treatment Trial) |
| ALCHEMIST | NCT02201992 | 2014 | Crizotinib in Treating Patients With Stage IB-IIIA Non-small Cell Lung Cancer That Has Been Removed by Surgery and ALK Fusion Mutations (An ALCHEMIST Treatment Trial) |
| ALCHEMIST | NCT02595944 | 2015 | Nivolumab After Surgery and Chemotherapy in Treating Patients With Stage IB-IIIA Non-small Cell Lung Cancer (An ALCHEMIST Treatment Trial) (ANVIL) |
| Bardia 2019 | Bardia 2018 | 2018 | Efficacy of sacituzumab govitecan (anti-trop- 2-sn-38 antibody-drug conjugate) for treatment-refractory hormone-receptor positive (hr+)/her2- metastatic breast cancer (mbc) |
| Basket of Basket | NCT03767075 | 2018 | A Modular Multi-Basket Trial to Improve Personalized Medicine in Cancer Patients (Basket of Baskets) (BoB) |
| BeGIN | NCT03872427 | 2019 | Testing Whether Cancers With Specific Mutations Respond Better to Glutaminase Inhibitor, CB-839 HCl, Anti-Cancer Treatment, BeGIN Study |
| BLU-667 Basket Trial | Iams 2018 | 2018 | Stop fretting the target: Next-generation ret inhibitors have arrived |
| BLU-667 Basket Trial | NCT03037385 | 2017 | Phase 1 Study of the Highly-selective RET Inhibitor BLU-667 in Patients With Thyroid Cancer, Non-Small Cell Lung Cancer, and Other Advanced Solid Tumors |
| bTMB-high basket trial | Nakamura 2019 | 2019 | Btmb-high basket trial: A multicenter phase ii trial of nivolumab monotherapy in patients with advanced gastrointestinal cancers with high blood tumor mutational burden (btmb) |
| bTMB-high basket trial | UMIN000033182 | 2018 | A multicenter phase II study of Nivolumab monotherapy in recurrent and/or metastatic gastrointestinal cancer patients with high Tumor Mutation Burden (TMB-H) |
| CAPTUR | NCT03297606 | 2017 | Canadian Profiling and Targeted Agent Utilization Trial (CAPTUR) (CAPTUR) |
| CAPTUR | Skamene 2018 | 2018 | Canadian profiling and targeted agent utilization trial (captur/pm.1): A phase ii basket precision medicine trial |
| CLUSTER | De Benedetti 2017A | 2017 | Efficacy and safety of canakinumab in patients with periodic fever syndromes (colchicine-resistant fmf, hids/mkd and traps): Results from a phase 3, pivotal, umbrella trial |
| CLUSTER | De Benedetti 2017B | 2017 | Pharmacokinetics and pharmacodynamics of canakinumab in patients with periodic fever syndromes (colchicine-resistant fmf, hids/mkd and traps): Results from a phase iii pivotal umbrella trial |
| CLUSTER | De Benedetti 2018 | 2018 | Canakinumab for the Treatment of Autoinflammatory Recurrent Fever Syndromes |
| CLUSTER | NCT02059291 | 2014 | Study of Efficacy and Safety of Canakinumab in Patients With Hereditary Periodic Fevers |
| CUSTOM | Lopez-Chavez 2015 | 2015 | Molecular profiling and targeted therapy for advanced thoracic malignancies: A biomarker-derived, multiarm, multihistology phase ii basket trial |
| CUSTOM | NCT01306045 | 2011 | Molecular Profiling and Targeted Therapy for Advanced Non-Small Cell Lung Cancer, Small Cell Lung Cancer, and Thymic Malignancies |
| Decoster 2018 | Decoster 2018 | 2018 | An explorative phase 2 study of afatinib for advanced cancers carrying an egfr, a her2 or a her3 mutation: A precision trial of the belgian society of medical oncology |
| Decoster 2018 | Precision 2: an open explorative phase II, open label study of afatinib in the treatment of advanced cancer carrying an EGFR, a HER2 or a HER3 mutation. | 2016 | Precision 2: an open explorative phase II, open label study of afatinib in the treatment of advanced cancer carrying an EGFR, a HER2 or a HER3 mutation. |
| Edenfield 2018 | Edenfield 2018 | 2018 | Improving care delivery for patients with rare cancers: A phase ii trial of durvalumab in combination with tremilumimab in subjects with advanced rare tumors in a large community health care system |
| FUZE Clinical Trial | NCT03834220 | 2019 | Basket Trial in Solid Tumors Harboring a Fusion of FGFR1, FGFR2 or FGFR3- (FUZE Clinical Trial) |
| Imatinib Target Exploration Consortium Study B2225 | Heinrich 2008 | 2008 | Phase II, Open-Label Study Evaluating the Activity of Imatinib in Treating Life-Threatening Malignancies Known to Be Associated with Imatinib-Sensitive Tyrosine Kinases |
| Imatinib Target Exploration Consortium Study B2225 | McArthur 2005 | 2005 | Molecular and Clinical Analysis of Locally Advanced Dermatofibrosarcoma Protuberans Treated With Imatinib: Imatinib Target Exploration Consortium Study B2225 |
| IMPACT 2 | Aletaha 2017 | 2017 | Disease activity states of the DAPSA, a psoriatic arthritis specific instrument, are valid against functional status and structural progression. |
| IMPACT 2 | NCT02152254 | 2014 | IMPACT 2: Randomized Study Evaluating Molecular Profiling and Targeted Agents in Metastatic Cancer |
| ITCC ESMART | NCT02813135 | 2016 | European Proof-of-Concept Therapeutic Stratification Trial of Molecular Anomalies in Relapsed or Refractory Tumors (ESMART) |
| K-Basket | NCT03017521 | 2017 | K-BASKET, TAS-117, PI3K/AKT Gene Aberration |
| K-Basket | NCT03491345 | 2018 | K-Basket, Avelumab, Biomarker-driven, Advanced Solid Tumor |
| LOXO-101: NAVIGATE, SCOUT | Chen 2018 | 2018 | Basket trial of trk inhibitors demonstrates efficacy in trk fusion-positive cancers |
| LOXO-101: NAVIGATE, SCOUT | Drilon 2018 | 2018 | Efficacy of Larotrectinib in TRK Fusion–Positive Cancers in Adults and Children |
| LOXO-101: NAVIGATE, SCOUT | NCT02122913 | 2014 | Oral TRK Inhibitor LOXO-101 for Treatment of Advanced Adult Solid Tumors |
| LOXO-101: NAVIGATE, SCOUT | NCT02576431 | 2015 | Study of LOXO-101 (Larotrectinib) in Subjects With NTRK Fusion Positive Solid Tumors (NAVIGATE) |
| LOXO-101: NAVIGATE, SCOUT | NCT02637687 | 2015 | Oral TRK Inhibitor LOXO-101 (Larotrectinib) for Treatment of Advanced Pediatric Solid or Primary Central Nervous System Tumors (SCOUT) |
| MEDIOLA | Bang 2019 | 2019 | An open-label, phase ii basket study of olaparib and durvalumab (mediola): Results in patients with relapsed gastric cancer |
| MEDIOLA | Domchek 2019 | 2019 | An open-label, phase ii basket study of olaparib and durvalumab (mediola): Updated results in patients with germline brca-mutated (gbrcam) metastatic breast cancer (mbc) |
| MEDIOLA | NCT02734004 | 2016 | A Phase I/II Study of MEDI4736 in Combination With Olaparib in Patients With Advanced Solid Tumors. (MEDIOLA) |
| MiMe-A | NCT03339843 | 2017 | Multiorgan Metabolic Imaging Response Assessment of Abemaciclib (MiMe-A) |
| MOVIE | NCT03518606 | 2018 | Metronomic Oral Vinorelbine Plus Anti-PD-L1/Anti-CTLA4 ImmunothErapy in Patients With Advanced Solid Tumours (MOVIE) |
| My Pathway | Hainsworth 2018 | 2018 | Targeted Therapy for Advanced Solid Tumors on the Basis of Molecular Profiles: Results From MyPathway, an Open-Label, Phase IIa Multiple Basket Study. |
| My Pathway | NCT02091141 | 2014 | My Pathway: A Study Evaluating Herceptin/Perjeta, Tarceva, Zelboraf/Cotellic, Erivedge, Alecensa, and Tecentriq Treatment Targeted Against Certain Molecular Alterations in Participants With Advanced Solid Tumors |
| NCI-MATCH | Barroilhet 2018 | 2018 | The NCI-MATCH trial and precision medicine in gynecologic cancers |
| NCI-MATCH | Chen 2016 | 2016 | NCI-Molecular Analysis for Therapy Choice (NCI-MATCH) trial: A novel public–private partnership |
| NCI-MATCH | Conley 2014 | 2014 | Molecular analysis for therapy choice: NCI MATCH. |
| NCI-MATCH | Moore 2016 | 2016 | Is the NCI MATCH trial a match for gynecologic oncology? |
| NCI-MATCH | NCT02465060 | 2015 | Targeted Therapy Directed by Genetic Testing in Treating Patients With Advanced Refractory Solid Tumors, Lymphomas, or Multiple Myeloma (The MATCH Screening Trial) |
| NCI-MPACT | NCT01827384 | 2013 | Molecular Profiling-Based Targeted Therapy in Treating Patients With Advanced Solid Tumors |
| NCI-MPACT | Nih 2016 | 2016 | Analytical Validation and Application of a Targeted Next-Generation Sequencing Mutation-Detection Assay for Use in Treatment Assignment in the NCI-MPACT Trial |
| NCT01876511 | Le 2015 | 2015 | PD-1 Blockade in Tumors with Mismatch-Repair Deficiency |
| NCT01876511 | NCT01876511 | 2013 | Phase 2 Study of MK-3475 in Patients With Microsatellite Unstable (MSI) Tumors |
| NCT02372006 | NCT02372006 | 2015 | Trial of Afatinib in Pediatric Tumours |
| NCT02399943 | NCT02399943 | 2015 | A Trial of Trametinib and Panitumumab in RAS/RAF Wild Type Advanced Colorectal Cancer |
| NCT02478320 | NCT02478320 | 2015 | Phase II Study of Ilorasertib (ABT348) in Patients With CDKN2A Deficient Solid Tumors |
| NCT02506517 | NCT02506517 | 2015 | A Study of Afatinib in Patients With Advanced Cancer With Changes in the HER Gene |
| NCT02938793 | NCT02938793 | 2016 | Durvalumab in Combination With Tremelimumab in Subjects With Advanced Rare Solid Tumors |
| NCT02955290 | NCT02955290 | 2016 | CIMAvax Vaccine, Nivolumab, and Pembrolizumab in Treating Patients With Advanced Non-small Cell Lung Cancer or Squamous Head and Neck Cancer |
| NCT03003195 | NCT03003195 | 2016 | accination of Patients With Metastatic Cancer |
| NCT03386721 | NCT03386721 | 2017 | Study to Evaluate the Therapeutic Activity of RO6874281 as a Combination Therapy in Participants With Advanced and/or Metastatic Solid Tumors |
| NCT03428802 | NCT03428802 | 2018 | Pembrolizumab in Treating Participants With Metastatic, Recurrent or Locally Advanced Cancer and Genomic Instability |
| NCT03525392 | NCT03525392 | 2018 | Platform Study for the Treatment of Relapsed or Refractory Aggressive Non-Hodgkin's Lymphoma (PRISM Study) |
| NCT03810872 | NCT03810872 | 2019 | An Explorative Study of Afatinib in the Treatment of Advanced Cancer Carrying an EGFR, a HER2 or a HER3 Mutation |
| NCT03982173 | NCT03982173 | 2019 | Basket Trial for Combination Therapy With Durvalumab (Anti-PDL1) (MEDI4736) and Tremelimumab (Anti-CTLA4) in Patients With Metastatic Solid Tumors (MATILDA) |
| Perez 2018 | NCT02454972 | 2015 | Clinical Trial of Lurbinectedin (PM01183) in Selected Advanced Solid Tumors |
| Perez 2018 | Perez 2018 | 2018 | Efficacy and safety of lurbinectedin (pm1183) in small cell lung cancer (sclc): Results from a phase 2 study |
| Perez 2018 | Subbiah 2018 | 2018 | Efficacy and safety of lurbinectedin (pm1183) in ewing sarcoma: Final results from a phase 2 study |
| PIK3CA Basket Trial | Hasegawa 2017 | 2017 | Phase ii basket trial of perifosine monotherapy for recurrent gynecologic cancer with or without pik3ca mutations |
| PM01183 Basket trial | Forster 2017 | 2017 | Activity of lurbinectedin (pm01183) as single agent and in combination in patients with endometrial cancer |
| PM01183 Basket trial | NCT01970540 | 2013 | Study Escalating Doses of PM01183 in Combination With Fixed Doxorubicin in Patients With Specific Advanced Unresectable Solid Tumors |
| RO5126766 (CH5127566) Basket Trial | Chenard-Poirier 2017 | 2017 | Results from the biomarker-driven basket trial of ro5126766 (ch5127566), a potent raf/mek inhibitor, in ras- or raf-mutated malignancies including multiple myeloma |
| RO5126766 (CH5127566) Basket Trial | Harris 2016 | 2016 | Updated efficacy and safety results from the phase I study of intermittent dosing of the dual MEK/RAF inhibitor, RO5126766 in patients (pts) with RAS/RAF mutated advanced solid tumours. |
| RO5126766 (CH5127566) Basket Trial | NCT02407509 | 2015 | Phase I Trial of RO5126766 Alone and in Combination With Everolimus (DDU RAF/MEK) |
| SHIVA | Le Tourneau 2015 | 2015 | Molecularly targeted therapy based on tumour molecular profiling versus conventional therapy for advanced cancer (SHIVA): a multicentre, open-label, proof-of-concept, randomised, controlled phase 2 trial. |
| SHIVA | NCT01771458 | 2013 | A Randomized Phase II Trial Comparing Therapy Based on Tumor Molecular Profiling Versus Conventional Therapy in Patients With Refractory Cancer (SHIVA) |
| Signature Program | NCT01831726 | 2013 | Dovitinib for Patients With Tumor Pathway Activations Inhibited by Dovitinib (SIGNATURE) |
| Signature Program | NCT01833169 | 2013 | BKM120 for Patients With PI3K-activated Tumors (SIGNATURE) |
| Signature Program | NCT01885195 | 2013 | MEK162 for Patients With RAS/RAF/MEK Activated Tumors (SIGNATURE) |
| Signature Program | NCT01981187 | 2013 | LGX818 for Patients With BRAFV600 Mutated Tumors (SIGNATURE) |
| Signature Program | NCT02002689 | 2013 | LDE225 for Patients With PTCH1 or SMO Mutated Tumors (SIGNATURE) |
| Signature Program | NCT02160041 | 2014 | BGJ398 for Patients With Tumors With FGFR Genetic Alterations (CBGJ398XUS04) |
| Signature Program | NCT02186821 | 2014 | Ceritinib (LDK378) for Patients Whose Tumors Have Aberrations in ALK or ROS1 (SIGNATURE) (SIGNATURE) |
| Signature Program | NCT02187783 | 2014 | LEE011 for Patients With CDK4/6 Pathway Activated Tumors (SIGNATURE) (SIGNATURE) |
| Signature Program | Slosberg 2018 | 2018 | Signature program: A platform of basket trials |
| SUMMIT | Hyman 2017A | 2017 | Neratinib + fulvestrant in erbb2-mutant, her2-non-amplified, estrogen receptor (ex positive, metastatic breast cancer (mbc): Preliminary analysis from the phase ii summit trial |
| SUMMIT | Hyman 2017B | 2017 | Neratinib in her2 or her3 mutant solid tumors: Summit, a global, multi-histology, open-label, phase 2 "basket" study |
| SUMMIT | NCT01953926 | 2013 | Neratinib HER Mutation Basket Study (SUMMIT) (SUMMIT) |
| TAPUR | NCT02693535 | 2016 | TAPUR: Testing the Use of Food and Drug Administration (FDA) Approved Drugs That Target a Specific Abnormality in a Tumor Gene in People With Advanced Stage Cancer (TAPUR) |
| TNT0009 Basket Trial | Derhaschnig 2016 | 2016 | Combined integrated protocol/basket trial design for a first-in-human trial |
| TNT0009 Basket Trial | Mühlbacher 2017 | 2017 | Blockade of HLA Antibody-Triggered Classical Complement Activation in Sera From Subjects Dosed With the Anti-C1s Monoclonal Antibody TNT009—Results from a Randomized First-in-Human Phase 1 Trial |
| TNT0009 Basket Trial | NCT02502903; EUDRA-CT2014-003881-26 | 2015 | Safety, Tolerability and Activity of TNT009 in Healthy Volunteers and Patients With Complement Mediated Disorders (TNT009-01) |
| VE-BASKET | Diamond 2018 | 2018 | Vemurafenib for braf v600-mutant erdheim-chester disease and langerhans cell histiocytosis analysis of data from the histology-independent, phase 2, open-label ve-basket study |
| VE-BASKET | Hyman 2015 | 2015 | Vemurafenib in Multiple Nonmelanoma Cancers with BRAF V600 Mutations |
| VE-BASKET | NCT01524978 | 2012 | A Study of Vemurafenib in Participants With BRAF V600 Mutation-Positive Cancers |
| Wainberg 2019 | NCT02034110 | 2014 | Efficacy and Safety of the Combination Therapy of Dabrafenib and Trametinib in Subjects With BRAF V600E- Mutated Rare Cancers |
| Wainberg 2019 | Wainberg 2019 | 2019 | Efficacy and safety of dabrafenib (d) and trametinib (t) in patients (pts) with braf v600e-mutated biliary tract cancer (btc): A cohort of the roar basket trial |

Table S7. List of included studies: Umbrella trials

| **Trial ID** | **Study ID** | **Year** | **Title** |
| --- | --- | --- | --- |
| **Umbrella trial** | | | |
| Bardia 2019 | Bardia 2019 | 2019 | Sacituzumab Govitecan-hziy in Refractory Metastatic Triple-Negative Breast Cancer. |
| Bardia 2019 | Cardillo 2015 | 2015 | Sacituzumab Govitecan (IMMU-132), an Anti-Trop-2/SN-38 Antibody-Drug Conjugate: Characterization and Efficacy in Pancreatic, Gastric, and Other Cancers. |
| Bardia 2019 | NCT01631552 | 2012 | Phase I/II Study of IMMU-132 in Patients With Epithelial Cancers |
| Bardia 2019 | Starodub 2015 | 2015 | First-in-Human Trial of a Novel Anti-Trop-2 Antibody-SN-38 Conjugate, Sacituzumab Govitecan, for the Treatment of Diverse Metastatic Solid Tumors. |
| BATTLE | Kim 2011 | 2011 | The BATTLE Trial: Personalizing Therapy for Lung Cancer |
| BATTLE | NCT00409968 | 2006 | BATTLE Program: Umbrella Protocol for Patients With Non-Small Cell Lung Cancer (NSCLC) |
| BATTLE | NCT00410059 | 2006 | BATTLE Program: Erlotinib in Previously Treated Subjects With Advanced NSCLC |
| BATTLE | NCT00410189 | 2006 | BATTLE Program: Erlotinib in Previously Treated Subjects With Advanced NSCLC |
| BATTLE | NCT00411632 | 2006 | BATTLE Program: Tarceva and Targretin in Patients With Advanced Non-Small Cell Lung Cancer (NSCLC) |
| BATTLE | NCT00411671 | 2006 | BATTLE Program: Sorafenib in Patients With NSCLC |
| BATTLE | Tam 2013 | 2013 | Feasibility of Image-Guided Transthoracic Core Needle Biopsy in the BATTLE Lung Trial |
| BATTLE-2 | NCT01248247 | 2010 | BATTLE-2 Program: A Biomarker-Integrated Targeted Therapy Study in Previously Treated Patients With Advanced Non-Small Cell Lung Cancer |
| BATTLE-2 | Papadimitrakopoulou 2015 | 2015 | The BATTLE-2 Study: A Biomarker-Integrated Targeted Therapy Study in Previously Treated Patients With Advanced Non–Small-Cell Lung Cancer |
| CheckMate 370 | NCT02574078 | 2015 | A Study of Nivolumab in Advanced Non-Small Cell Lung Cancer (NSCLC) (CheckMate370) |
| CheckMate 370 | Spiegel 2018 | 2018 | Phase 1/2 Study of the Safety and Tolerability of Nivolumab Plus Crizotinib for the First-Line Treatment of Anaplastic Lymphoma Kinase Translocation — Positive Advanced Non–Small Cell Lung Cancer (CheckMate 370) |
| FOCUS4 | Adams 2018 | 2018 | Inhibition of egfr, her2, and her3 signalling in patients with colorectal cancer wild-type for braf, pik3ca, kras, and nras (focus4-d): A phase 2-3 randomised trial |
| FOCUS4 | FOCUS4 2018 | 2018 | FOCUS4-N Protocol A randomised controlled comparison of capecitabine against active monitoring after first-line treatment for patients with colorectal cancer |
| FOCUS4 | ISRCTN90061546 | 2013 | FOCUS4: Molecular selection of therapy in colorectal cancer |
| FOCUS4 | Kaplan 2013 | 2013 | Evaluating many treatments and biomarkers in oncology: a new design. |
| FOCUS4 | Kaplan 2015 | 2015 | The focus4 design for biomarker stratified trials |
| FOCUS4 | Richman 2017 | 2017 | Focus4: Mams trial design in action. Early closure of focus4-d (pan-her 1, 2 and 3 inhibitor versus placebo) in advanced colorectal cancer (acrc) patients, with tumours wildtype (wt) for kras, nras, braf and pik3ca |
| FUTURE | NCT03805399 | 2019 | FUSCC Refractory TNBC Umbrella (FUTURE) (FUTURE) |
| Genomics Enabled Medicine for Melanoma | LoRusso 2015 | 2015 | Pilot Trial of Selecting Molecularly-Guided Therapy for Patients with non-V600 BRAF Mutant Metastatic Melanoma: Experience of the SU2C/MRA Melanoma Dream Team |
| Genomics Enabled Medicine for Melanoma | NCT02094872 | 2014 | My Pathway: A Study Evaluating Herceptin/Perjeta, Tarceva, Zelboraf/Cotellic, Erivedge, Alecensa, and Tecentriq Treatment Targeted Against Certain Molecular Alterations in Participants With Advanced Solid Tumors |
| LUNG-MAP | Edelman 2017 | 2017 | A phase ii study of palbociclib (p) for previously treated cell cycle gene alteration positive patients (pts) with stage iv squamous cell lung cancer (scc): Lung-map sub-study swog s1400c |
| LUNG-MAP | Ferrarotto 2015 | 2015 | Lung-map--framework, overview, and design principles |
| LUNG-MAP | Herbst 2015 | 2015 | Lung master protocol (lung-map)-a biomarker-driven protocol for accelerating development of therapies for squamous cell lung cancer: Swog s1400 |
| LUNG-MAP | NCT02154490 | 2014 | Lung-MAP: Biomarker-Targeted Second-Line Therapy in Treating Patients With Recurrent Stage IV Squamous Cell Lung Cancer |
| LUNG-MAP | NCT02766335 | 2016 | Lung-MAP: Durvalumab as Second-Line Therapy in Treating Patients With Recurrent Stage IV Squamous Cell Lung Cancer and No Matching Biomarkers |
| LUNG-MAP | NCT02785913 | 2016 | S1400B Lung-MAP: Taselisib as Second-Line Therapy in Treating Patients With Recurrent Stage IV Squamous Cell Lung Cancer and Positive Biomarker Matches |
| LUNG-MAP | NCT02785939 | 2016 | Lung-MAP: Palbociclib as Second-Line Therapy in Treating Cell Cycle Gene Alteration Positive Patients With Recurrent Stage IV Squamous Cell Lung Cancer |
| LUNG-MAP | Papadimitrakopoulou 2017A | 2017 | A phase ii study of durvalumab (medi4736) for previously treated patients with stage iv squamous nsclc (sqnsclc): Lung-map substudy swog s1400a |
| LUNG-MAP | Papadimitrakopoulou 2017B | 2017 | Lung-map (s1400) lung master protocol: Accrual and genomic screening updates |
| LUNG-MAP | Papadimitrakopoulou 2018 | 2018 | First comprehensive report of impact of genomic alterations, chemotherapy, targeted therapy and immunotherapy on outcomes in the genomics driven squamous master protocol lungmap |
| LUNG-MAP | Wade 2017 | 2017 | A phase ii study of gdc-0032 (taselisib) for previously treated pi3k positive patients with stage iv squamous cell lung cancer (sqnsclc): Lung-map substudy swog s1400b |
| MASTER KEY | Okuma 2018 | 2018 | Master key project: A basket/umbrella trial for rare cancers in japan |
| MASTER KEY | UMIN000027552 | 2017 | A prospective clinical registry study of genetic profiling and targeted therapies in patients with rare cancers; MASTER KEY Protocol |
| National Lung Matrix Trial | ISRCTN38344105 | 2015 | National lung matrix: multi-drug phase II trial in non-small cell (NSC) lung cancer |
| National Lung Matrix Trial | Middleton 2015 | 2015 | The national lung matrix trial: Translating the biology of stratification in advanced non-small-cell lung cancer |
| National Lung Matrix Trial | NCT02664935 | 2016 | National Lung Matrix Trial: Multi-drug Phase II Trial in Non-Small Cell Lung Cancer |
| NCT Neuro Master Match (N2M2) | [NCT03158389](https://clinicaltrials.gov/ct2/show/NCT03158389) | 2017 | NCT Neuro Master Match - N²M² (NOA-20) (N²M²) |
| NCT Neuro Master Match (N2M2) | Wick 2019 | 2019 | N 2 m 2 (noa-20) phase i/ii trial of molecularly matched targeted therapies plus radiotherapy in patients with newly diagnosed non-mgmt hypermethylated glioblastoma |
| NCT02951091 | NCT02951091 | 2016 | Biomarker-Integrated Umbrella, Advanced Gastric Cancer |
| NCT03267589 | NCT03267589 | 2017 | Trial in Patients With Relapsed Ovarian Cancer |
| NCT03356587 | NCT03356587 | 2017 | A Biomarker-driven, Open Label, Single Arm, Multicentre Phase II Study of Abemaciclib in Patients With Recurrent or Metastatic Head and Neck Squamous Cell Carcinoma Who Failed to Platinum-based Therapy |
| plasmaMATCH | NCT03182634 | 2017 | The UK Plasma Based Molecular Profiling of Advanced Breast Cancer to Inform Therapeutic CHoices (plasmaMATCH) Trial (plasmaMATCH) |
| plasmaMATCH | Turner 2018 | 2018 | The plasmaMATCH trial: A multiple parallel cohort, open­label, multi­centre phase II clinical trial of ctDNA screening to direct targeted therapies in patients with advanced breast cancer (CRUK/15/010) |
| Precision Panc: PRIMUS | Graham 2018 | 2018 | Primus-001: An adaptive phase ii study of folfox-a (folfox and nab-paclitaxel) versus ag (nab-paclitaxel and gemcitabine) in patients with metastatic pancreatic cancer, with integrated biomarker evaluation (isrctn75002153) - part of precision-panc |
| Precision Panc: PRIMUS | ISRCTN14879538 | 2018 | Precision Panc: Advancing personalised medicine treatment strategies for pancreatic cancer |
| Precision Panc: PRIMUS | ISRCTN75002153 | 2017 | PRIMUS001: A study looking at two different treatments for pancreatic cancer that has spread to other parts of the body |
| Precision Panc: PRIMUS | Valle 2019 | 2019 | Precision-panc master protocol: Personalizing treatment for pancreatic cancer isrctn14879538-part of precision-panc united kingdom |
| TRIUMPH | NCT03292250 | 2017 | Korean Cancer Study Group: Translational bIomarker Driven UMbrella Project for Head and Neck (TRIUMPH), Esophageal Squamous Cell Carcinoma- Part 1 (HNSCC)] |
| TRUMP | NCT03574402 | 2018 | Phase II Umbrella Study Directed by Next Generation Sequencing (TRUMP) |
| UPSTREAM | Galot 2018 | 2018 | Eortc 1559-hncg: A pilot study of personalized biomarker-based treatment strategy or immunotherapy in patients with recurrent/metastatic (r/m) squamous cell carcinoma of the head and neck (scchn) -"upstream" |
| UPSTREAM | NCT03088059 | 2017 | Biomarker-based Study in R/M SCCHN (UPSTREAM) |

Table S8. List of included studies: Platform trials

| **Trial ID** | **Study ID** | **Year** | **Title** |
| --- | --- | --- | --- |
| **Platform trials** | | | |
| ALIC4E | Butler 2018 | 2018 | A trial like alic4e: Why design a platform, response-adaptive, open, randomised controlled trial of antivirals for influenza-like illness? |
| ALIC4E | ISRCTN27908921 | 2015 | Antivirals for influenza-like illness? Are they effective? |
| CREATE | NCT01524926 | 2012 | CREATE: Cross-tumoral Phase 2 With Crizotinib (CREATE) |
| CREATE | Peron 2019 | 2019 | A multinational, multi-tumour basket study in very rare cancer types: The european organization for research and treatment of cancer phase ii 90101 'create' trial |
| CREATE | Schoffski 2017 | 2017 | Activity and safety of crizotinib in patients with advanced clear-cell sarcoma with MET alterations: European Organization for Research and Treatment of Cancer phase II trial 90101 ‘CREATE’ |
| CREATE | Schoffski 2018 | 2018 | Crizotinib in patients with advanced, inoperable inflammatory myofibroblastic tumours with and without anaplastic lymphoma kinase gene alterations (European Organisation for Research and Treatment of Cancer 90101 CREATE): a multicentre, single-drug, prospective, non-randomised phase 2 trial. |
| CREATE | Schoffski 2018B | 2018 | Activity and safety of crizotinib in patients with alveolar soft part sarcoma with rearrangement of TFE3: European Organization for Research and Treatment of Cancer (EORTC) phase II trial 90101 ‘CREATE’ |
| CREATE | Schoffski 2018C | 2018 | The tyrosine kinase inhibitor crizotinib does not have clinically meaningful activity in heavily pre-treated patients with advanced alveolar rhabdomyosarcoma with FOXO rearrangement: European Organisation for Research and Treatment of Cancer phase 2 trial 90101 'CREATE'. |
| EBOLA | Berry 2016 | 2016 | A response adaptive randomization platform trial for efficient evaluation of ebola virus treatments: A model for pandemic response |
| EBOLA | NCT02380625 | 2015 | Multiple Treatments for Ebola Virus Disease (EVD) |
| FRACTION | NCT02750514 | 2016 | An Investigational Immuno-therapy Study to Test Combination Treatments in Patients With Advanced Non-Small Cell Lung Cancer (FRACTION-Lung) |
| FRACTION | NCT02935634 | 2016 | A Study to Test Combination Treatments in Patients With Advanced Gastric Cancer (FRACTION-GC) |
| FRACTION | NCT02996110 | 2016 | A Study to Test Combination Treatments in People With Advanced Renal Cell Carcinoma (FRACTION-RCC) |
| FRACTION | Simonsen 2018 | 2018 | The fast real-time assessment of combination therapies in immuno-oncology (fraction) program: Innovative, high-throughput clinical screening of immunotherapies |
| GBM AGILE | Alexander 2018 | 2018 | Adaptive Global Innovative Learning Environment for Glioblastoma: GBM AGILE |
| GBM AGILE | Berry 2017 | 2017 | Adaptively randomized seamless-phase multiarm platform trial: Glioblastoma multiforme adaptive global innovative learning environment (gbm agile) |
| GBM AGILE | NCT03970447 | 2019 | A Trial to Evaluate Multiple Regimens in Newly Diagnosed and Recurrent Glioblastoma (GBM AGILE) |
| GBM AGILE | Wen 2018 | 2018 | Adaptive global innovative learning environment for glioblastoma: Gbm agile |
| I-SPY2 | Barker 2009 | 2009 | I-spy 2: An adaptive breast cancer trial design in the setting of neoadjuvant chemotherapy |
| I-SPY2 | Forero 2017 | 2017 | Efficacy of hsp90 inhibitor ganetespib plus standard neoadjuvant therapy in high-risk breast cancer: Results from the i-spy 2 trial |
| I-SPY2 | Nanda 2017 | 2017 | Pembrolizumab plus standard neoadjuvant therapy for high-risk breast cancer (bc): Results from i-spy 2 |
| I-SPY2 | NCT01042379 | 2010 | I-SPY 2 TRIAL: Neoadjuvant and Personalized Adaptive Novel Agents to Treat Breast Cancer (I-SPY 2) |
| I-SPY2 | Paoloni 2017 | 2017 | A longitudinal look at toxicity management within a platform trial: Lessons from the i-spy 2 trial |
| I-SPY2 | Park 2016 | 2016 | Adaptive randomization of neratinib in early breast cancer |
| I-SPY2 | Rugo 2016 | 2016 | Adaptive randomization of veliparib-carboplatin treatment in breast cancer |
| I-SPY2 | Symmans 2018 | 2018 | Residual cancer burden (rcb) as prognostic in the i-spy 2 trial |
| I-SPY2 | Yau 2019 | 2019 | Expression-based immune signatures as predictors of neoadjuvant targeted-/chemo-therapy response: Experience from the i-spy 2 trial of 1000 patients across 10 therapies |
| I-SPY2 | Yee 2017 | 2017 | The evaluation of ganitumab/metformin plus standard neoadjuvant therapy in high-risk breast cancer: Results from the ispy 2 trial |
| INSIGhT | Alexander 2017 | 2017 | Individualized screening trial of innovative glioblastoma therapy (INSIGhT). |
| INSIGhT | NCT02977780 | 2016 | INdividualized Screening Trial of Innovative Glioblastoma Therapy (INSIGhT) |
| LEAP | NCT03092674 | 2017 | Azacitidine With or Without Nivolumab or Midostaurin, or Decitabine and Cytarabine Alone in Treating Older Patients With Newly Diagnosed Acute Myeloid Leukemia or High-Risk Myelodysplastic Syndrome |
| LeAP | Walter 2018 | 2018 | Intergroup leap trial (s1612): A randomized phase 2/3 platform trial to test novel therapeutics in medically less fit older adults with acute myeloid leukemia |
| NCT03739710 | NCT03739710 | 2018 | Phase II Platform Trial of Novel Regimens Versus Standard of Care (SoC) in Non-small Cell Lung Cancer (NSCLC) |
| PLATforM | NCT03484923 | 2018 | Study of Efficacy and Safety of Novel Spartalizumab Combinations in Patients With Previously Treated Unresectable or Metastatic Melanoma (PLATforM) |
| PLATforM | Weber 2019 | 2019 | A randomized, open-label, phase 2, open-platform study evaluating the efficacy and safety of novel spartalizumab (pdr001) combinations in previously treated unresectable or metastatic melanoma (platform) |
| PLATO | ISRCTN88455282 | 2016 | PLATO - Personalising anal cancer radiotherapy dose |
| PLATO | Sebag-Montefiore 2017 | 2017 | Optimising rt dose for anal cancer-the development of three clinical trials in one platform |
| PRISM | NCT03527147 | 2018 | Study to Evaluate the Safety and Activity (Including Distribution) of 177Lu-3BP-227 in Subjects With Solid Tumours Expressing Neurotensin Receptor Type 1. |
| RAMPART | NCT03288532 | 2017 | Renal Adjuvant MultiPle Arm Randomised Trial (RAMPART) |
| REMAP-CAP | NCT02735707 | 2016 | Randomized, Embedded, Multifactorial Adaptive Platform Trial for Community- Acquired Pneumonia (REMAP-CAP) |
| REMAP-CAP | Rahmani 2018 | 2018 | Components and considerations of a critical care trial: A remap-cap substudy |
| STAMPEDE | Gilson 2017 | 2017 | Incorporating biomarker stratification into stampede: An adaptive multi-arm, multi-stage trial platform |
| STAMPEDE | Grist 2018 | 2018 | Implementing molecular characterisation of prostate cancer tissue from patients recruited to the multi-centre stampede trial: The stratosphere consortium |
| STAMPEDE | ISRCTN78818544 | 2004 | Systemic therapy in advanced or metastatic prostate cancer: evaluation of drug efficacy |
| STAMPEDE | James 2009 | 2009 | Systemic therapy for advancing or metastatic prostate cancer (stampede): A multi-arm, multistage randomized controlled trial |
| STAMPEDE | James 2011 | 2011 | Celecoxib plus hormone therapy vs hormone therapy alone for hormone-sensitive prostate cancer: First results from the stampede randomised controlled trial (mrc pr08) |
| STAMPEDE | James 2012 | 2012 | Celecoxib plus hormone therapy versus hormone therapy alone for hormone-sensitive prostate cancer: First results from the stampede multiarm, multistage, randomised controlled trial |
| STAMPEDE | James 2017A | 2017 | Abiraterone for prostate cancer not previously treated with hormone therapy |
| STAMPEDE | James 2017B | 2017 | Adding abiraterone for men with high-risk prostate cancer (pca) starting long-term androgen deprivation therapy (adt): Survival results from stampede (nct00268476) |
| STAMPEDE | James 2017C | 2017 | Adding abiraterone for patients (pts) with high-risk prostate cancer (pca) starting long-term androgen deprivation therapy (adt): Outcomes in non-metastatic (m0) patients from stampede (nct00268476) |
| STAMPEDE | Mason Md 2017 | 2017 | Adding celecoxib with or without zoledronic acid for hormone-naive prostate cancer: Long-term survival results from an adaptive, multiarm, multistage, platform, randomized controlled trial |
| STAMPEDE | NCT00268476 | 2005 | Systemic Therapy in Advancing or Metastatic Prostate Cancer: Evaluation of Drug Efficacy (STAMPEDE) |
| STAMPEDE | Sydes 2009 | 2009 | Issues in applying multi-arm multi-stage methodology to a clinical trial in prostate cancer: The mrc stampede trial |
| STAMPEDE | Sydes 2011 | 2011 | Flexible trial design in practice - dropping and adding arms in stampede: A multi-arm multi-stage randomised controlled trial |
| STAMPEDE | Sydes 2012 | 2012 | Flexible trial design in practice - stopping arms for lack-of-benefit and adding research arms mid-trial in stampede: A multi-arm multi-stage randomized controlled trial |
| STAMPEDE | Sydes 2017 | 2017 | Pr adding abiraterone acetate plus prednisolone (aap) or docetaxel for patients (pts) with high-risk prostate cancer (pca) starting long-term androgen deprivation therapy (adt): Directly randomised data from stampede (nct00268476) |
| STAMPEDE | Sydes 2018 | 2018 | Adding abiraterone or docetaxel to long-term hormone therapy for prostate cancer: Directly randomised data from the stampede multi-arm, multi-stage platform protocol |
| The DIAN-TU platform | Bateman 2017 | 2017 | The dian-tu next generation alzheimer's prevention trial: Adaptive design and disease progression model |
| The DIAN-TU platform | NCT01760005 | 2013 | Dominantly Inherited Alzheimer Network Trial: An Opportunity to Prevent Dementia. A Study of Potential Disease Modifying Treatments in Individuals at Risk for or With a Type of Early Onset Alzheimer's Disease Caused by a Genetic Mutation. (DIAN-TU) |
| UPMC REMAP | NCT03861767 | 2019 | REMAP Trial for Optimizing Surgical Outcomes at UPMC (UPMC REMAP) |

1. Trial and disease characteristics of master protocols and their geographic location

Table S9. Trial characteristics of master protocols

| **Master protocol ID** | **Registry** | **Trial status** | **Phase** | **Randomized (Y/N)** | **Masking** | **Total N** | **Proposed trial duration (mo)** | **Intervention types** | **No of intervention** | **Control: Y/N** |
| --- | --- | --- | --- | --- | --- | --- | --- | --- | --- | --- |
| **Basket trials** | | | | | | | | | | |
| AcSe´ | NCT02304809 | Recruiting | II | No | Open label | 500 | 90.9 | Drugs | 1 | No |
| Ado-trastuzumab Basket trial | NCT02675829 | Recruiting | IIA | No | Open label | 100 | 35.9 | Drugs | 1 | No |
| AGADIR | NCT03915678 | Not yet recruiting | II | No | Open label | 247 | 47.9 | Drugs | 1 | No |
| ALCHEMIST | NCT02201992; NCT02193282; NCT02595944 | Recruiting | III | Yes | Open label | 1542 | 118.8 | Drugs | 3 | Yes |
| Bardia 2019 | NCT01631552 | Active, not recruiting | I/II | No | Open label | 500 | 81.8 | Drugs | 1 | No |
| Basket of Basket | NCT03767075 | Recruiting | II | No | Open label | 1000 | 4.0 | Drugs | 1 | No |
| BeGIN | NCT03872427 | Not yet recruiting | II | No | Open label | 108 | 20.0 | Drugs | 1 | No |
| BLU-667 Basket Trial | NCT03037385 | Recruiting | I | No | Open label | 250 | 71.8 | Drugs | 1 | No |
| bTMB-high basket trial | UMIN000033182 | Recruiting | II | No | Open label | 70 | -- | Drugs | 1 | No |
| CAPTUR | NCT03297606 | Recruiting | II | No | Open label | 720 | 46.9 | Drugs | 13 | No |
| CLUSTER | NCT02059291 | Completed/published | III | Yes | Open label | 203 | 36.9 | Drugs | 3 | Yes |
| CREATE | NCT01524926 | Active, not recruiting | II | No | Open label | 582 | 12.0 | Drugs | 1 | No |
| CUSTOM | NCT01306045 | Recruiting/published | II | No | Open label | 600 | 105.7 | Drugs | 5 | No |
| Decoster 2018 | EudraCT No: 2016-003411-34 | Recruiting | II | No | Open label | NR | -- | Drugs | 2 | No |
| Edenfield 2018 | NCT02938793 | Recruiting | II | No | Open label | 50 | 59.9 | Drugs | 1 | No |
| ESMART | NCT02813135 | Recruiting | I/II | No | Open label | 397 | 66.9 | Drugs | 6 | No |
| FUZE Clinical Trial | NCT03834220 | Recruiting | II | No | Open label | 125 | 41.0 | Drugs | 1 | No |
| Imatinib Target Exploration Consortium Study B2225. | NR | Completed/published | II | No | Open label | 186 | 45.9 | Drugs | 1 | No |
| IMPACT 2 | NCT02152254 | Active, not recruiting | I/II | Yes | Single blind  (investigator) | 391 | 71.9 | Drugs | NR | Yes |
| K-Basket | NCT03491345; NCT03017521 | Recruiting | II | No | Open label | 90 | 39.9 | Drugs | 2 | No |
| LOXO-101 NAVIGATE SCOUT | NCT02122913 | Recruiting | I/II | No | Open label | 90 | 58.9 | Drugs | 1 | No |
| MEDIOLA | NCT02734004 | Recruiting | I/II | No | Open label | 427 | 76.5 | Drugs | 2 | No |
| MiMe-A | NCT03339843 | Recruiting | II | No | Open label | 85 | 33.9 | Drugs | 1 | No |
| MOVIE | NCT03518606 | Recruiting | I/II | No | Open label | 150 | 61.9 | Drugs | 1 | No |
| My Pathway | NCT02091141 | Recruiting/published | II | No | Open label | 765 | 83.8 | Drugs | 6 | No |
| NCI-MATCH | NCT02465060 | Recruiting | II | No | Open label | 6452 | 81.8 | Drugs | 21 | No |
| NCI-MPACT | NCT01827384 | Recruiting | II | No | Single blind | 700 | 64.8 | Drugs | 6 | No |
| NCT01876511 | -- | Recruiting | II | No | Open label | 171 | 95.8 | Drugs | 1 | No |
| NCT02372006 | -- | Recruiting | I/II | No | Open label | 55 | 51.9 | Drugs | 1 | No |
| NCT02399943 | -- | Recruiting | II | No | Open label | 26 | 65.9 | Drugs | 1 | No |
| NCT02478320 | -- | Active, not recruiting | II | No | Open label | 12 | 71.8 | Drugs | 1 | No |
| NCT02506517 | -- | Recruiting | II | No | Open label | 30 | 39.9 | Drugs | 1 | No |
| NCT02955290 | NCT02955290 | Recruiting | I/II | No | Open label | 181 | 53.9 | Drugs and vaccine | 2 | No |
| NCT03003195 | -- | Proposed | II | No | Open label | 80 | 41.9 | Vaccine | 1 | No |
| NCT03386721 | -- | Recruiting | II | No | Open label | 360 | 34.9 | Drugs | 3 | No |
| NCT03428802 | -- | Recruiting | II | No | Open label | 40 | 31.0 | Drugs | 1 | No |
| NCT03525392 | -- | Recruiting | I/II | No | Open label | 320 | 45.9 | Drugs | 1 | No |
| NCT03810872 | NCT03810872 | Recruiting | II | No | Open label | 87 | 65.9 | Drugs | 1 | No |
| NCT03982173 | NCT03982173 | Active, not recruiting | II | No | Open label | 88 | 47.9 | Drugs | 1 | No |
| Perez 2018 | NCT02454972 | Active, not recruiting | II | No | Open label | 345 | 52.9 | Drugs | 1 | No |
| PM01183 Basket trial | NCT01970540 | Completed/published | I | No | Open label | 122 | 71.9 | Drugs | 1 | No |
| RO5126766 (CH5127566) Basket Trial | NCT02407509 | Recruiting | I | No | Open label | 94 | 96.8 | Drugs | 2 | No |
| SHIVA | NCT01771458 | Completed/published | II | Yes | Open label | 742 | 46.9 | Drugs | 9 | Yes |
| Signature Program | NCT01833169; NCT01831726; NCT01885195; NCT01981187; NCT02002689; NCT02160041; NCT02186821; NCT02187783 | Completed/published | II | No | Open label | 596 | 60.9 | Drugs | 8 | No |
| SUMMIT | NCT01953926 | Recruiting | II | No | Open label | 392 | 100.8 | Drugs | 4 | No |
| TAPUR | NCT02693535 | Recruiting | II | No | Open label | 1440 | 35.9 | Drugs | 14 | No |
| TNT0009 Basket Trial | NCT02502903; EUDRA-CT2014-003881-26 | Recruiting/published | I | Yes | Double blind | 98 | 39.9 | Drugs | 3 | Yes |
| VE-BASKET | NCT01524978 | Completed/published | II | No | Open label | 208 | 68.9 | Drugs | 2 | No |
| Wainberg 2019 | NCT02034110 | Active, not recruiting | II | No | Open label | 206 | 75.4 | Drugs | 1 | No |
| **Umbrella trials** | | | | | | | | | | |
| BATTLE-1 | NCT00409968 | Active, not recruiting (results published) | II | Yes | Open label | 250 | 167.7 | Drugs | 4 | No |
| BATTLE-2 | NCT01248247 | Active, not recruiting (results published) | II | Yes | Open label | 334 | 95.8 | Drugs | 4 | No |
| CheckMate 370 | NCT02574078 | Active, not recruiting | I/II | Yes | Open label | 342 | 41.3 | Drugs | 5 | Yes |
| FOCUS4 | ISRCTN90061546 | Completed/published | II/III | Yes | Double blind | 384 | 58.8 | Drugs | 5 | Yes |
| FUTURE | NCT03805399 | Recruiting | Ib/II | No | Open label | 140 | 46.9 | Drugs | 7 | No |
| Genomics Enabled Medicine for Melanoma | NCT02094872 | Active, not recruiting | II | Yes | Open label | 47 | 46.9 | Drugs | 19 | Yes |
| LUNG-MAP | NCT02154490 | Recruiting | II/III | Yes | Open label | 10000 | 93.8 | Drugs | 5 | Yes |
| MASTER KEY | UMIN000027552 | Recruiting | NR | NR | NR | 1000 | 95.8 | Drugs | NR | NR |
| National Lung Matrix Trial | NCT02664935 | Recruiting | II | No | Open label | 620 | 75.9 | Drugs | 8 | No |
| NCT Neuro Master Match (N2M2) | [NCT03158389](https://clinicaltrials.gov/ct2/show/NCT03158389) | Recruiting | I/IIa | No | Open label | 350 | 76.7 | Drugs | 7 | No |
| NCT02951091 | -- | Recruiting | II | Yes | Open label | 400 | 52.9 | Drugs | 4 | Yes |
| NCT03267589 | -- | Recruiting | II | Yes (part 2) | Open label | 75 | 62.9 | Drugs | 3 | Yes (2nd stage) |
| NCT03356587 | -- | Recruiting | II | No | Open label | 32 | 37.9 | Drugs | 5 | No |
| plasmaMATCH | NCT03182634 | Recruiting | IIA | No | Open label | 1000 | 82.8 | Drugs | 4 | No |
| Precision Panc: PRIMUS | ISRCTN75002153 | Recruiting | II | Yes | Open label | 5000 | 44.9 | Drugs | 2+ | Yes |
| TRIUMPH | NCT03292250 | Recruiting | II | No | Open label | 259 | 38.9 | Drugs | 5 | No |
| TRUMP | NCT03574402 | Not yet recruiting | II | No | Open label | 400 | 76.9 | Drugs | 5 | No |
| UPSTREAM | NCT03088059 | Recruiting | II | No | Open label | 340 | 48.4 | Drugs | 7 | No |
| **Platform trials** | | | | | | | | | | |
| ALIC4E | ISRCTN27908921 | Completed | "NA" | Yes | Open label | 675 | 39.0 | Drugs | NR | Yes |
| EBOLA | NCT02380625 | Terminated | II | Yes | Open label | 150 | 35.9 | Drugs | 3 | Yes |
| FRACTION | NCT02935634; NCT02750514; NCT02996110 | Recruiting | II | Yes | Open label | 1004 | 68.3 | Drugs | 5+ | Yes |
| GBM AGILE | NR | Recruiting | II/III | Yes | NR | NR | -- | Drugs | NR | Yes |
| I-SPY2 | NCT01042379 | Recruiting/published | II | Yes | Open label | 1920 | 128.8 | Drugs | 18 | Yes |
| INSIGhT | NCT02977780 | Recruiting | II | Yes | Open label | 280 | 50.8 | Drugs | 3 | Yes |
| LEAP | NCT03092674 | Suspended (Unscheduled safety data review) | II/III | Yes | Open label | 1670 | 67.8 | Drugs | 4+ | Yes |
| NCT03739710 | NCT03739710 | Recruiting | II | Yes | Open label | 105 | 37.9 | Drugs | 1+ | Yes |
| PLATforM | NCT03484923 | Recruiting | II | Yes | Open label | 230 | 32.6 | Drugs | 4 | No |
| PLATO | ISRCTN88455282 | Recruiting | II | Yes (part 2) | NR | 892 | 58.9 | Drugs, radiation, surgery | 5 | No |
| PRISM | NCT03527147 | Recruiting | I | No | Open label | 42 | 31.0 | Drugs | 3+ | No |
| RAMPART | NCT03288532 | Recruiting | III | Yes | Open label | 1750 | 232.6 | Drugs | 2 | Yes |
| REMAP-CAP | NCT02735707 | Recruiting | III | Yes | Open label | 6800 | 73.8 | Drugs | 8 | No |
| STAMPEDE | NCT00268476; ISRCTN78818544 | Recruiting/published | II/III | Yes | Open label | 12200 | 229.6 | Drugs/Radiation | 10 | Yes |
| The DIAN-TU platform | NCT01760005 | Active, not recruiting | II/III | Yes | Double blind | 438 | 131.7 | Drugs | 3 | Yes |
| UPMC REMAP | NCT03861767 | Recruiting | III | Yes | Triple blind | 2000 | 15.0 | Drugs | 9+ | Yes |

Table S10. Disease characteristics of master protocols

| **Master protocol ID** | **Disease area** | **Type of population** | **Age eligibility** | **Number of diseases** | **Key eligibility criteria for stratification** | **Number of subgroups** |
| --- | --- | --- | --- | --- | --- | --- |
| **Basket trials** | | | | | | |
| AcSe´ | Oncology | Adults | 18yr or older | 11+ | Biomarkers | 11 |
| Ado-trastuzumab Basket trial | Oncology | Adults | 18yr or older | 4+ | Biomarkers | 4 |
| AGADIR | Oncology | Adults | 18 yrs or older | 6 | Tumour type | 6 |
| ALCHEMIST | Oncology | Adults | 18yr or older | 1 | Biomarkers and treatment history | 3 |
| Bardia 2019 | Oncology | Adults | 18yr or older | 16 | Biomarker | 16+ |
| Basket of Basket | Oncology | Adults | 18 yrs or older | 2+ | Biomarkers | 6 |
| BeGIN | Oncology | Mixed | 12 years or older | 4+ | Biomarkers | 4+ |
| BLU-667 Basket Trial | Oncology | Adults | 18yr or older | 4+ | Biomarkers and treatment history | 6 |
| bTMB-high basket trial | Oncology | Adults | 20 yrs or older | 6 | Biomarkers | NR |
| CAPTUR | Oncology | Adults | 18yr or older | 3 | BIomarkers | 14 |
| CLUSTER | Hereditary  Periodic  Fevers | Mixed | 1mo or older | 3 | Disease type | 3 |
| CREATE | Oncology | Mixed | At least 1yr | 6 | Biomarkers and tumour type | 6 |
| CUSTOM | Oncology | Adults | 18yr or older | 3 | Biomarkers | 6 |
| Decoster 2018 | Oncology | NR | NR | NR | Biomarkers | NR |
| Edenfield 2018 | Oncology | Adults | 18 yrs or older | 2+ | NR | 2+ |
| ESMART | Oncology | Pediatrics &  adolescents | <18yr | 2+ | Biomarkers | 6 |
| FUZE Clinical Trial | Oncology | Adults | 18 yrs or older | 2+ | Biomarkers | NR |
| Imatinib Target Exploration Consortium Study B2225. | Oncology | Mixed | 15yr or older | 23 | Biomarkers | 3 |
| IMPACT 2 | Oncology | Adults | 18yr or older | NR | Biomarkers and tumour type | NR |
| K-Basket | Oncology | Adults | 19 or older | 1+ | Biomarkers | 2+ |
| LOXO-101 NAVIGATE SCOUT | Oncology | Adults | 18yr or older | NR | Biomarkers and tumour type | NR |
| MEDIOLA | Oncology | Adults | 18-130 years | 4 | Biomarkers and Tumour type | 10 |
| MiMe-A | Oncology | Adults | 18yr or older | 5 | Biomarkers | 5 |
| MOVIE | Oncology | Adults | 18yr or older | 5+ | Biomarkers and treatment history | 5 |
| My Pathway | Oncology | Adults | 18yr or older | NR | Biomarkers | 6 |
| NCI-MATCH | Oncology | Adults | 18yr or older | 49 | Biomarkers | 27 |
| NCI-MPACT | Oncology | Adults | 18yr or older | NR | Biomarkers | NR |
| NCT01876511 | Oncology | Adults | 18yr or older | 2+ | Biomarkers | 4 |
| NCT02372006 | Oncology | Pediatrics | 1-18yr | 8+ | Biomarkers and treatment history | NR |
| NCT02399943 | Oncology | Adults | 18yr or older | 1 | Biomarkers and treatment history | NR |
| NCT02478320 | Oncology | Adults | 18yr or older | NR | Biomarkers and tumour type | NR |
| NCT02506517 | Oncology | Adults | 18yr or older | NR | Biomarkers | NR |
| NCT02955290 | Oncology | Adults | 18 yrs or older | 2+ | Treatment history | 4 |
| NCT03003195 | Oncology | Adults | 18yr or older | NR | Tumour type | NR |
| NCT03386721 | Oncology | Adults | 18yr or older | 4 | Biomarkers and treatment history | 10 |
| NCT03428802 | Oncology | Adults | 18yr or older | 3+ | Biomarkers | 2+ |
| NCT03525392 | Oncology | Adults | 18yr or older | 5 | Biomarkers | 5 |
| NCT03810872 | Oncology | Adults | 18 yrs or older | 2+ | Biomarkers | 3+ |
| NCT03982173 | Oncology | Adults | 18 yrs or older | 5+ | Biomarkers and treatment history | 5 |
| Perez 2018 | Oncology | Adults | 18 yrs or older | NR | NR | NR |
| PM01183 Basket trial | Oncology | Adults | 18-75yr | 10+ | Treatment history and tumour types | 3 |
| RO5126766 (CH5127566) Basket Trial | Oncology | Adults | 18yr or older | 2+ | Biomarkers | 4 |
| SHIVA | Oncology | Adults | 18yr or older | 26 | Biomarkers | 10 |
| Signature Program | Oncology | Adults | 18yr or older | 3+ | Biomarkers | NR |
| SUMMIT | Oncology | Adults | 18yr or older | 2+ | Biomarkers | 4 |
| TAPUR | Oncology | Mixed | 12yr or older | 3+ | Biomarkers | 14 |
| TNT0009 Basket Trial | Complement-Mediated Disorders | Adults | 18yr or older | 4 | Biomarkers | 3 |
| VE-BASKET | Oncology | Mixed | 16yr or older | 6+ | Biomarkers | 7 |
| Wainberg 2019 | Oncology | Adults | 18 yrs or older | 9 | Biomarkers | NR |
| **Umbrella trials** | | | | | | |
| BATTLE-1 | Oncology | Adults | 18yr or older | 1 | Biomarkers and tumour type | 4 |
| BATTLE-2 | Oncology | Adults | 18yr or older | 1 | Biomarkers and tumour type | NR |
| CheckMate 370 | Oncology | Adults | 18yr or older | 1 | Biomarkers | NR |
| FOCUS4 | Oncology | Adults | 18yr or older | 1 | Biomarkers and tumour type | 5 |
| FUTURE | Oncology | Adults | 18 - 75 years | 1 | Biomarkers | 4 |
| Genomics Enabled Medicine for Melanoma | Oncology | Adults | 18yr or older | 1 | Biomarkers | 19 |
| LUNG-MAP | Oncology | Adults | 18yr or older | 1 | Biomarkers and tumour type | 5 |
| MASTER KEY | Oncology | Mixed | 1 years or older | 2+ | Biomarkers | NR |
| National Lung Matrix Trial | Oncology | Adults | 18yr or older | 1 | Biomarkers and tumour type | 8 |
| NCT Neuro Master Match (N2M2) | Oncology | Adults | 18 yrs or older | 1 | Biomarkers | NR |
| NCT02951091 | Oncology | Adults | 19 or older | 1 | Biomarkers | 4 |
| NCT03267589 | Oncology | Adults | 18yr or older | 1 | Biomarkers | 3 |
| NCT03356587 | Oncology | Adults | 20yr or older | 1 | Biomarkers | 5 |
| plasmaMATCH | Oncology | Adults | 18yr or older | 1 | Biomarkers | 4 |
| Precision Panc: PRIMUS | Oncology | Mixed | 16 years or older | 1 | Biomarkers | NR |
| TRIUMPH | Oncology | Adults | 20yr or older | 1 | Biomarkers | 5 |
| TRUMP | Oncology | Adults | 18yr or older | 1 | Biomarkers | 5 |
| UPSTREAM | Oncology | Adults | 18 yrs or older | 1 | Biomarkers | NR |
| **Platform trials** | | | | | | |
| ALIC4E | Influenza | Mixed | At least 1yr | 1 | Disease type and risk factors | NR |
| EBOLA | Ebola | Mixed | 6mo or older | 1 | Disease type | NR |
| FRACTION | Oncology | Adults | 18 yrs or older | 3 | Biomarkers | 12 |
| GBM AGILE | Oncology | NR | NR | 1 | Biomarkers and tumour type | 6 |
| I-SPY2 | Oncology | Adults | 18yr or older | 1 | Biomarkers and tumour type | 3 |
| INSIGhT | Oncology | Adults | 18yr or older | 1 | Biomarkers and tumour type | 4 |
| LEAP | Oncology | Adults (older) | 60yr or older | 1 | Biomarkers | NR |
| NCT03739710 | Oncology | Adults | 18 yrs or older | 1 | NR | 2+ |
| PLATforM | Oncology | Adults | 18 yrs or older | 1 | NR | NR |
| PLATO | Oncology | Mixed | 16yr or older | 1 | NR | NR |
| PRISM | Oncology | Adults | 18yr or older | 1 | NR | NR |
| RAMPART | Oncology | Adults | 18 yrs or older | 1 | NR | NR |
| REMAP-CAP | Pneumonia | Adults | 18yr or older | 1 | NR | NR |
| STAMPEDE | Oncology | Adults | 18yr or older | 1 | Biomarkers and tumour type | 5 |
| The DIAN-TU platform | Alzheimer's | Adults | 18-80yr | 3 | Biomarkers | NR |
| UPMC REMAP | Surgery | Adults | 18 yrs or older | 1 | NR | NR |

Table S11. Geographic location of master protocols

| **Master protocol ID** | **N of centers involved** | **LMICs involved** | **Countries** |
| --- | --- | --- | --- |
| **Basket trials** | | | |
| AcSe´ | NR | No | France |
| Ado-trastuzumab Basket trial | 1 | No | US |
| AGADIR | 10 | No | France |
| ALCHEMIST | 1349 | No | US |
| Bardia 2019 | 12 | No | US |
| Basket of Basket | 3 | No | France, Spain, and UK |
| BeGIN | 1 | No | US |
| BLU-667 Basket Trial | 30 | No | US, France, Italy, South Korea, Singapore, Spain, Taiwan, and UK |
| bTMB-high basket trial | NR | No | Japan |
| CAPTUR | 5 | No | Canada |
| CLUSTER | 65 | No | US, Belgium, Canada, France, Germany, Hungary, Ireland, Israel, Italy, Japan, Netherlands, Russia, Spain, Switzerland, Turkey, UK |
| CREATE | 25 | No | Belgium, France, Germany, Italy, Netherlands, Norway, Poland, Slovakia, Slovenia, UK |
| CUSTOM | 1 | No | US |
| Decoster 2018 | NR | No | Belgium |
| Edenfield 2018 | 1 | No | US |
| ESMART | 6 | No | France, Germany, Italy, Netherlands, Spain, and UK |
| FUZE Clinical Trial | 28 | No | US, Czechia, Greece, Singapore, and Spain |
| Imatinib Target Exploration Consortium Study B2225. | 13 | No | Australia, US, UK, Finland, Canada, Switzerland, Belgium |
| IMPACT 2 | 1 | No | US |
| K-Basket | 1 | No | South Korea |
| LOXO-101 NAVIGATE SCOUT | 8 | No | US |
| MEDIOLA | 53 | No | US, France, Israel, Republic of Korea, Netherlands, Switzerland, UK |
| MiMe-A | 1 | No | Belgium |
| MOVIE | 3 | No | France |
| My Pathway | 50 | No | US |
| NCI-MATCH | 1253 | No | US |
| NCI-MPACT | 7 | No | US |
| NCT01876511 | 7 | No | US |
| NCT02372006 | 30 | No | US, Australia, Austria, Canada, Denmark, Faroe Islands, France, Germany, Italy, Netherlands, Spain, UK |
| NCT02399943 | 1 | No | Canada |
| NCT02478320 | 1 | No | US |
| NCT02506517 | 1 | No | Canada |
| NCT02955290 | 1 | No | US |
| NCT03003195 | 1 | No | US |
| NCT03386721 | 45 | No | US, Belgium, Israel, South Korea, Netherlands, New Zealand, Poland, Russia, Singapore, Spain, Taiwan, Turkey, UK |
| NCT03428802 | 1 | No | US |
| NCT03525392 | 1 | No | France |
| NCT03810872 | 5 | No | Belgium |
| NCT03982173 | 1 | No | France |
| Perez 2018 | 38 | No | US, Belgium, France, Germany, Italy, Spain, Sweden, Switzerland, UK |
| PM01183 Basket trial | 7 | No | Spain, UK |
| RO5126766 (CH5127566) Basket Trial | 2 | No | UK |
| SHIVA | 8 | No | France |
| Signature Program | 69 | No | US |
| SUMMIT | 44 | No | UK, Spain, Republic of Korea, Italy, Isreal, Ireland, Ireland, France, Finland, Denmark, Canada, Belgium, Australia, US |
| TAPUR | 24 | No | US |
| TNT0009 Basket Trial | 1 | No | Austria |
| VE-BASKET | 34 | Yes (China) | UK, Spain, Germany, France, China, US, |
| Wainberg 2019 | 52 | No | US, Austria, Belgium, Canada, Denmark, France, Germany, Italy, Japan, Republic of Korea, Netherlands, Norway, Spain, Sweden |
| **Umbrella trials** | | | |
| BATTLE-1 | 1 | No | US |
| BATTLE-2 | 2 | No | US |
| CheckMate 370 | 133 | No | US |
| FOCUS4 | 18 | No | UK |
| FUTURE | 1 | Yes (China) | China |
| Genomics Enabled Medicine for Melanoma | 8 | No | US |
| LUNG-MAP | 1004 | No | US, Canada |
| MASTER KEY | NR | No | Japan |
| National Lung Matrix Trial | 20 | No | UK |
| NCT Neuro Master Match (N2M2) | 13 | No | Germany |
| NCT02951091 | 1 | No | South Korea |
| NCT03267589 | 5 | No | Denmark, Finland, Norway |
| NCT03356587 | 1 | No | South Korea |
| plasmaMATCH | 8 | No | UK |
| Precision Panc: PRIMUS | 12 | No | UK |
| TRIUMPH | 1 | No | South Korea |
| TRUMP | NR | Yes (China) | China |
| UPSTREAM | 30 | No | Belgium, France, Italy, UK |
| **Platform trials** | | | |
| ALIC4E | 39 | No | Belgium, Croatia, Czech Republic, Denmark, France, Greece, Hungary, Ireland, Lithuania, Netherlands, Norway, Poland, Spain, Sweden, Switzerland, United Kingdom |
| EBOLA | NR | Yes (West Africa) | Guinea, Sierra Leone, Liberia |
| FRACTION | 50 | No | US, Australia, Austria, Canada, France, Italy, Spain, Switzerland |
| GBM AGILE | NR | Yes (China) | US, Australia, China |
| I-SPY2 | 24 | No | US |
| INSIGhT | 10 | No | US |
| LEAP | 508 | No | US |
| NCT03739710 | 64 | No | US, Canada, France, Germany, Italy, South Korea, Netherlands, Poland, Romania, Russia, Spain, Sweden |
| PLATforM | 32 | No | US, Australia, Canada, France, Germany, Italy, Netherlands, Spain, Switzerland, UK |
| PLATO | 47 | No | UK |
| PRISM | 6 | No | US |
| RAMPART | 5 | No | UK |
| REMAP-CAP | 36 | No | Australia, New Zealand |
| STAMPEDE | 120 | No | UK, Switzerland |
| The DIAN-TU platform | 36 | Yes (Brazil and Mexico) | US, Argentina, Australia, Brazil, Canada, France, Germany, Ireland, Italy, Mexico, Puerto Rico, Spain, UK |
| UPMC REMAP | 1 | No | US |

Reference:

1. Abrams J, Conley B, Mooney M, Zwiebel J, Chen A, Welch JJ, Takebe N, Malik S, McShane L, Korn E, et al: **National Cancer Institute's Precision Medicine Initiatives for the new National Clinical Trials Network.** *Am Soc Clin Oncol Educ Book* 2014**:**71-76.

2. Bando H, Takebe N: **Perspectives on research activity in the USA on Cancer Precision Medicine.** *Jpn J Clin Oncol* 2016, **46:**106-110.

3. Beckman RA, Antonijevic Z, Kalamegham R, Chen C: **Adaptive Design for a Confirmatory Basket Trial in Multiple Tumor Types Based on a Putative Predictive Biomarker.** *Clin Pharmacol Ther* 2016, **100:**617-625.

4. Berry DA: **The Brave New World of clinical cancer research: Adaptive biomarker-driven trials integrating clinical practice with clinical research.** *Mol Oncol* 2015, **9:**951-959.

5. Berry SM, Connor JT, Lewis RJ: **The platform trial: an efficient strategy for evaluating multiple treatments.** *JAMA* 2015, **313:**1619-1620.

6. Blumenthal GM, Bunn PA, Jr., Chaft JE, McCoach CE, Perez EA, Scagliotti GV, Carbone DP, Aerts H, Aisner DL, Bergh J, et al: **Current Status and Future Perspectives on Neoadjuvant Therapy in Lung Cancer.** *J Thorac Oncol* 2018, **13:**1818-1831.

7. Bratton DJ, Phillips PP, Parmar MK: **A multi-arm multi-stage clinical trial design for binary outcomes with application to tuberculosis.** *BMC Med Res Methodol* 2013, **13:**139.

8. Butler CC, Connor JT, Lewis RJ, Broglio K, Saville BR, Cook J, van der Velden A, Verheij T: **Answering patient-centred questions efficiently: response-adaptive platform trials in primary care.** *Br J Gen Pract* 2018, **68:**294-295.

9. Chen Y, Chi P: **Basket trial of TRK inhibitors demonstrates efficacy in TRK fusion-positive cancers.** *Journal of Hematology and Oncology* 2018, **11:**78.

10. Collignon O, Koenig F, Koch A, Hemmings RJ, Petavy F, Saint-Raymond A, Papaluca-Amati M, Posch M: **Adaptive designs in clinical trials: from scientific advice to marketing authorisation to the European Medicine Agency.** *Trials* 2018, **19:**642.

11. Cunanan KM, Iasonos A, Shen R, Begg CB, Gonen M: **An efficient basket trial design.** *Stat Med* 2017, **36:**1568-1579.

12. **Master Protocols: Efficient Clinical Trial Design Strategies to Expedite Development of Oncology Drugs and Biologics Guidance for Industry (Draft Guidance)** [<https://www.fda.gov/downloads/Drugs/GuidanceComplianceRegulatoryInformation/Guidances/UCM621817.pdf>]

13. **Adaptive Designs for Clinical Trials of Drugs and Biologics Guidance for Industry (Draft Guidance)** [<https://www.fda.gov/downloads/drugs/guidances/ucm201790.pdf>]

14. Fountzilas E, Tsimberidou AM: **Overview of precision oncology trials: challenges and opportunities.** *Expert Rev Clin Pharmacol* 2018, **11:**797-804.

15. Grignolo A, Siu A: **Improving Drug Development and Patient Access With the Right People, Processes, and Culture: What Needs to Happen Right Now to Bring Better Medicines to the Patients Who Need Them.** *Therapeutic innovation & regulatory science* 2019, **53:**398-402.

16. Heckman-Stoddard BM, Smith JJ: **Precision medicine clinical trials: defining new treatment strategies.** *Semin Oncol Nurs* 2014, **30:**109-116.

17. Herbst RS, Gandara DR, Hirsch FR, Redman MW, LeBlanc M, Mack PC, Schwartz LH, Vokes E, Ramalingam SS, Bradley JD, et al: **Lung Master Protocol (Lung-MAP)-A Biomarker-Driven Protocol for Accelerating Development of Therapies for Squamous Cell Lung Cancer: SWOG S1400.** *Clinical cancer research : an official journal of the American Association for Cancer Research* 2015, **21:**1514-1524.

18. Hirakawa A, Asano J, Sato H, Teramukai S: **Master protocol trials in oncology: Review and new trial designs.** *Contemp Clin Trials Commun* 2018, **12:**1-8.

19. Hobbs BP, Landin R: **Bayesian basket trial design with exchangeability monitoring.** *Stat Med* 2018, **37:**3557-3572.

20. Hobbs BP, Chen N, Lee JJ: **Controlled multi-arm platform design using predictive probability.** *Statistical methods in medical research* 2018, **27:**65-78.

21. Iams WT, Lovly CM: **Stop fRETting the Target: Next-Generation RET Inhibitors Have Arrived.** *Cancer discovery* 2018, **8:**797-799.

22. Khan T, Stewart M, Blackman S, Rousseau R, Donoghue M, Cohen K, Seibel N, Fleury M, Benettaib B, Malik R, et al: **Accelerating Pediatric Cancer Drug Development: Challenges and Opportunities for Pediatric Master Protocols.** *Ther Innov Regul Sci* 2018**:**2168479018774533.

23. Kourie HR, Aoun F: **Why balls are not put inside the basket? A reflection on testicular cancer clinical trial design.** *Investigational new drugs* 2016, **34:**513-514.

24. Lam VK, Papadimitrakopoulou V: **Master protocols in lung cancer: experience from Lung Master Protocol.** *Curr Opin Oncol* 2018, **30:**92-97.

25. Ledford H: **'Master protocol' aims to revamp cancer trials: pilot project will bring drug companies together to test targeted lung-cancer therapies.** *Nature* 2013, **498:**146-148.

26. Lee J: **Biomarker-based Bayesian adaptive designs for targeted agent development.** In *Journal of Thoracic Oncology*. 2011: S91-S92.

27. Lih CJ, Takebe N: **Considerations of developing an NGS assay for clinical applications in precision oncology: The NCI-MATCH NGS assay experience.** *Curr Probl Cancer* 2017, **41:**201-211.

28. Lin J, Bunn V: **Comparison of multi-arm multi-stage design and adaptive randomization in platform clinical trials.** *Contemp Clin Trials* 2017, **54:**48-59.

29. Manca C, Hill C, Hujer AM, Patel R, Evans SR, Bonomo RA, Kreiswirth BN, Laboratory Center of the Antibacterial Resistance Leadership G: **Leading Antibacterial Laboratory Research by Integrating Conventional and Innovative Approaches: The Laboratory Center of the Antibacterial Resistance Leadership Group.** *Clin Infect Dis* 2017, **64:**S13-S17.

30. Mandrekar SJ, Dahlberg SE, Simon R: **Improving Clinical Trial Efficiency: Thinking outside the Box.** *Am Soc Clin Oncol Educ Book* 2015**:**e141-147.

31. Martin A: **Using Trial Infrastructure Efficiently: Platform, Factorial, And Umbrella Trial Designs.** In *Asia-Pacific Journal of Clinical Oncology*. 2017: 39-39.

32. McCabe M: **SP-0590: Novel developments in paediatric cancer.** *Radiotherapy and Oncology* 2017, **123:**S310-S311.

33. Mullard A: **NCI-MATCH trial pushes cancer umbrella trial paradigm.** *Nat Rev Drug Discov* 2015, **14:**513-515.

34. Ornes S: **Core Concept: Basket trial approach capitalizes on the molecular mechanisms of tumors.** *Proceedings of the National Academy of Sciences* 2016, **113:**7007-7008.

35. Parmar MK, Sydes MR, Cafferty FH, Choodari-Oskooei B, Langley RE, Brown L, Phillips PP, Spears MR, Rowley S, Kaplan R: **Testing many treatments within a single protocol over 10 years at MRC Clinical Trials Unit at UCL: Multi-arm, multi-stage platform, umbrella and basket protocols.** *Clinical Trials* 2017, **14:**451-461.

36. Phillips PP, Gillespie SH, Boeree M, Heinrich N, Aarnoutse R, McHugh T, Pletschette M, Lienhardt C, Hafner R, Mgone C, et al: **Innovative trial designs are practical solutions for improving the treatment of tuberculosis.** *J Infect Dis* 2012, **205 Suppl 2:**S250-257.

37. Pitts PJ, Le Louet H, Katz G: **21st-Century Oncology Drug Safety via New-Age Regulatory Standards and Practices.** *Ther Innov Regul Sci* 2018**:**2168479018809689.

38. Redman MW, Allegra CJ: **The Master Protocol Concept.** *Semin Oncol* 2015, **42:**724-730.

39. Renfro LA, Sargent DJ: **Statistical controversies in clinical research: basket trials, umbrella trials, and other master protocols: a review and examples.** *Ann Oncol* 2017, **28:**34-43.

40. Saville BR, Berry SM: **Efficiencies of platform clinical trials: A vision of the future.** *Clin Trials* 2016, **13:**358-366.

41. Simon R: **Genomic Alteration-Driven Clinical Trial Designs in Oncology.** *Ann Intern Med* 2016, **165:**270-278.

42. Steuer CE, Papadimitrakopoulou V, Herbst RS, Redman MW, Hirsch FR, Mack PC, Ramalingam SS, Gandara DR: **Innovative Clinical Trials: The LUNG-MAP Study.** *Clin Pharmacol Ther* 2015, **97:**488-491.

43. Szczykutowicz TP, Bour RK, Rubert N, Wendt G, Pozniak M, Ranallo FN: **CT protocol management: simplifying the process by using a master protocol concept.** *J Appl Clin Med Phys* 2015, **16:**228-243.

44. Tanguturi SK, Trippa L, Ramkissoon SH, Pelton K, Knoff D, Sandak D, Lindeman NI, Ligon AH, Beroukhim R, Parmigiani G, et al: **Leveraging molecular datasets for biomarker-based clinical trial design in glioblastoma.** *Neuro Oncol* 2017, **19:**908-917.

45. Tao JJ, Schram AM, Hyman DM: **Basket Studies: Redefining Clinical Trials in the Era of Genome-Driven Oncology.** *Annu Rev Med* 2018, **69:**319-331.

46. Teo MY, O'Shaughnessy MJ, McBride SM, Vargas HA, Scher HI: **Drug development for noncastrate prostate cancer in a changed therapeutic landscape.** *Nat Rev Clin Oncol* 2018, **15:**150.

47. Tolles J, Lewis RJ: **Adaptive and platform trials in remote damage control resuscitation.** *J Trauma Acute Care Surg* 2018, **84:**S28-S34.

48. Trusheim MR, Shrier AA, Antonijevic Z, Beckman RA, Campbell RK, Chen C, Flaherty KT, Loewy J, Lacombe D, Madhavan S, et al: **PIPELINEs: Creating Comparable Clinical Knowledge Efficiently by Linking Trial Platforms.** *Clin Pharmacol Ther* 2016, **100:**713-729.

49. Ventz S, Barry WT, Parmigiani G, Trippa L: **Bayesian response-adaptive designs for basket trials.** *Biometrics* 2017, **73:**905-915.

50. Walter RB, Michaelis LC, Othus M, Uy GL, Radich JP, Little RF, Hita S, Saini L, Foran JM, Gerds AT, et al: **Intergroup LEAP trial (S1612): A randomized phase 2/3 platform trial to test novel therapeutics in medically less fit older adults with acute myeloid leukemia.** *Am J Hematol* 2018, **93:**E49-E52.

51. Woodcock J, LaVange LM: **Master protocols to study multiple therapies, multiple diseases, or both.** *New England Journal of Medicine* 2017, **377:**62-70.

52. Zardavas D, Piccart-Gebhart M: **Clinical trials of precision medicine through molecular profiling: focus on breast cancer.** *Am Soc Clin Oncol Educ Book* 2015, **35:**e183-190.
